# Adapted Live SARS‐CoV‐2 Vaccine Elicits Rapid Mucosal Immunity, Protects From Disease, and Reduces Shedding of XBB.1.5

**DOI:** 10.1002/eji.70196

**Published:** 2026-05-04

**Authors:** Jana Kochmann, Tobias Britzke, Nico Joël Halwe, Lorenz Ulrich, Angele Breithaupt, G. Tuba Barut, Nadine Ebert, Bettina Salome Trüeb, Volker Thiel, Anca Dorhoi, Martin Beer, Donata Hoffmann, Björn Corleis, Jacob Schön

**Affiliations:** ^1^ Institute of Immunology Friedrich‐Loeffler‐Institut Greifswald‐Insel Riems Germany; ^2^ Department of Experimental Animal Facilities and Biorisk Management Friedrich‐Loeffler‐Institut Greifswald‐Insel Riems Germany; ^3^ Institute of Diagnostic Virology Friedrich‐Loeffler‐Institut Greifswald‐Insel Riems Germany; ^4^ Institute of Virology and Immunology Bern and Mittelhäusern Bern Switzerland; ^5^ Department of Infectious Diseases and Pathobiology Vetsuisse Faculty University of Bern Bern Switzerland; ^6^ Multidisciplinary Center for Infectious Diseases University of Bern Bern Switzerland; ^7^ European Virus Bioinformatics Center Jena Germany

## Abstract

The emergence of SARS‐CoV‐2 variants, like XBB.1.5, causing immune evasion and frequent breakthrough infections, emphasizes the need for vaccines that limit transmission and target newly emerging variants. Mucosal vaccines, particularly live attenuated vaccines (LAV), are promising candidates for inducing strong mucosal immune responses to prevent viral replication and transmission. Vaccination with the previously described “one‐to‐stop” codon‐modified LAV OTS‐228, carrying the ancestral spike protein, induced sterilizing immunity against ancestral SARS‐CoV‐2 but also broad protection against Omicron variants, including XBB.1.5, but transmission of XBB.1.5 to contacts could not be prevented completely. As a proof‐of‐concept, we updated OTS‐228 by replacing the sequence coding for the ancestral SARS‐CoV‐2 spike protein with that of the XBB.1.5 variant. We applied flow cytometry to detect SARS‐CoV‐2‐specific T cell responses, as well as ELISA and qPCR, to characterize systemic and mucosal immune responses in Syrian hamsters in detail. The new OTS construct designated as “OTS‐300” exhibited an optimal safety profile in Syrian hamsters comparable to the original candidate vaccine. A single‐dose intranasal (i.n.) vaccination with OTS‐300 protects against disease, substantially limits XBB.1.5 replication, and reduces transmission in Syrian hamsters, showcasing the adaptability of the OTS platform for other emerging variants. OTS‐300 induced accelerated mucosal and systemic antibody responses and reduced virus‐mediated inflammation as compared with an intramuscularly delivered mRNA vaccine encoding the XBB.1.5 Spike.

## Graphical abstract

An attenuated live vaccine (OTS‐300) expressing the XBB.1.5 Spike provided rapid and complete protection against homologous challenge in the hamster model, accompanied by fast systemic and mucosal antibody responses and markedly reduced transmission compared with XBB.1.5 mRNA vaccination. Therefore, vaccination with the OTS LAV platform may help to disrupt transmission chains.

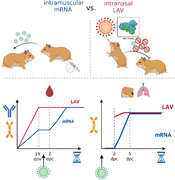

## 1 Introduction

Continuous emergence of SARS‐CoV‐2 variants of concern (VOCs) harboring substantial numbers of mutations in the spike protein resulted in antigenic shift and thereby waning efficacy of previously acquired neutralizing antibodies [1, 2, 3]. As a consequence of antibody escape and altered transmissibility, breakthrough infections occur frequently, as observed for the SARS‐CoV‐2 variant XBB.1.5 [1, 2, 4, 5]. This highlights the urgent need for a vaccine that substantially limits breakthrough infections due to new viral variants. An ideal vaccine should elicit local, immediate, and effective immune responses in the upper respiratory tract, the site of SARS‐CoV‐2 entry, to prevent viral replication and transmission. Reducing virus transmission is essential to slow down the emergence of new strains. However, vaccines approved for intramuscular (i.m.) vaccination against SARS‐CoV‐2 appear to induce less potent mucosal immunity in the upper respiratory tract compared with mucosal vaccines [6, 7, 8, 9]. Efforts to deliver vaccines intranasally (i.n.) showed that live attenuated viruses (LAV) are promising platforms for mucosal vaccination [10, 11, 12, 13, 14].

We previously described a highly efficient and attenuated SARS‐CoV‐2 LAV candidate “OTS‐228” based on the ancestral SARS‐CoV‐2 virus [10, 15]. The attenuated phenotype of this vaccine is achieved by several hundred synonymous codon changes in open reading frame (ORF) 1ab, so‐called “one‐to‐stop” codons, which increase the chance of premature termination codons [16]. In addition, functional knock‐out of nonstructural protein 1 (nsp1), as well as the deletion of ORF6‐8 and the spike polybasic cleavage site (PCS), were used for further attenuation of OTS‐228 [10]. Here, we demonstrate that OTS‐228 can be used as a platform technology by incorporating spike protein sequences from the latest VOCs. As proof of concept, we integrated the Omicron XBB.1.5 spike sequence in the OTS‐228 backbone and tested this construct, designated as “OTS‐300” in Syrian hamsters.

Although Syrian hamsters are commonly used as an animal model for SARS‐CoV‐2, tools to monitor immune responses in Syrian hamsters are not well established yet [17, 18, 19, 20, 21]. Consequently, a detailed understanding of their immune responses to vaccination and infection remains limited. To not only evaluate the OTS‐300 vaccine candidate but also to fill this gap in knowledge, we aimed to characterize immune responses in Syrian hamsters in greater detail. We evaluated mucosal and systemic immune responses in this species after OTS‐300 and mRNA vaccination over time. We applied enzyme‐linked immunosorbent assay (ELISA) to detect SARS‐CoV‐2‐specific IgA, flow cytometry to ascertain SARS‐CoV‐2‐specific T cells, and real‐time quantitative polymerase chain reaction (qPCR) to assess virus‐induced inflammation.

Intranasal vaccination is considered a promising strategy against respiratory viruses. It may induce superior protection compared with parenteral vaccination [17, 22] by eliciting mucosal antibodies and tissue‐resident lymphocytes in the respiratory tract, both known correlates of protection against SARS‐CoV‐2 [23, 24, 25]. Single‐dose vaccination in naïve Syrian hamsters with OTS‐300 was safe and protected against disease after subsequent challenge infection with Omicron XBB.1.5. Moreover, OTS‐300 vaccination also substantially reduced viral transmission. Detailed analyses in Syrian hamsters indicated that reduced transmission was likely driven by rapid mucosal and systemic immune responses after i.n. vaccination.

## 2 Results

### 2.1 Update of OTS‐228 Vaccine Backbone With the XBB.1.5 Spike

**FIGURE 1:**
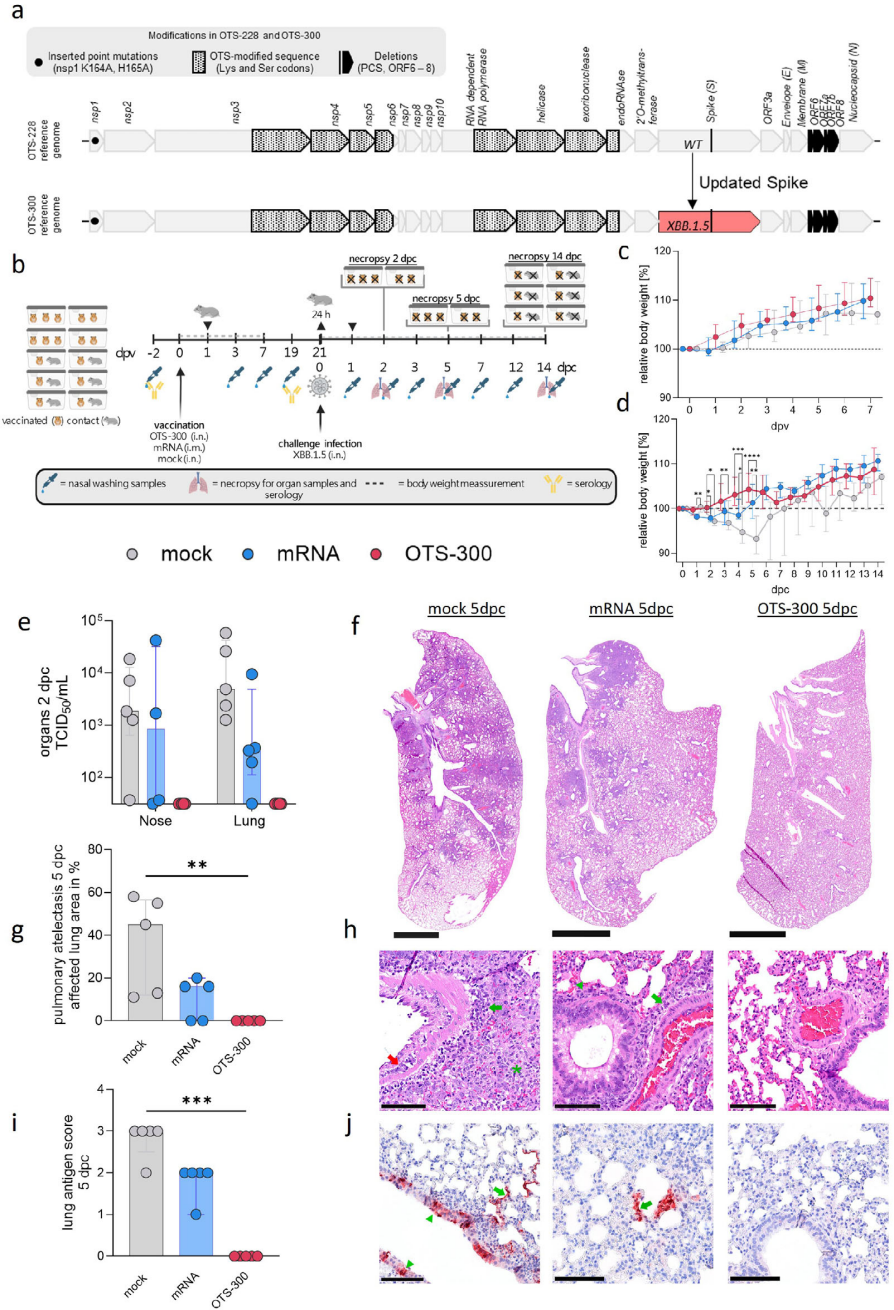
The SARS‐CoV‐2 OTS‐300 vaccine expressing the XBB.1.5 spike protein is safe and shows high efficacy against challenge infection in Syrian hamsters. (a) OTS‐300 includes several attenuating mutations (nsp1^K164A, H165A^, ORF6‐8 deletions, S1/S2 cleavage site deletion, and stop codon modifications in nsp3‐6 and the replication complex) and incorporates the XBB.1.5 spike sequence. (b) Experimental setup: Syrian hamsters (*n* = 16/group) were vaccinated with OTS‐300 (70 µL, 10^4^ tissue culture infectious dose 50% (TCID_50_)) i.n., Comirnaty Omicron XBB.1.5 mRNA vaccine (5 µg in 50 µL) i.m. or cell culture medium (70 µL, mock). Challenge infection was performed by i.n. inoculation with Omicron XBB.1.5 (70 µL 10^4,5^ TCID_50_) 21 dpv. Naive contact animals (*n* = 6/group) were co‐housed in a 1:1 ratio 1 dpv and separated right before challenge for one day. Overview created with BioRender. (c) Relative body weight (median with interquartile range) post vaccination (*n* = 16) (d) or postchallenge infection. (e) Individual and median SARS‐CoV‐2 XBB.1.5 TCID_50_ per mL with interquartile range in organ samples titrated by plaque assay on Vero E6 cells two days post challenge (dpc) (*n* = 5). (f–j) Histopathology at 5 dpc: (f) Hematoxylin–eosin‐stained lung sections showed pneumonia‐related atelectasis in mRNA‐ or nonimmunized control hamsters but not in OTS‐300‐immunized hamsters, bar 2.5 mm. (g) Lung evaluation for pneumonia‐related atelectasis, shown as a percentage of the affected area with median and interquartile range. (h) Representative detail images of hematoxylin–eosin‐stained lung sections showed perivascular (green arrow) and peribronchial (green arrowhead) inflammatory infiltrates, vasculitis (red arrow), alveolar edema, and alveolar immune cell infiltration (green asterisk) in mRNA‐ and mock‐immunized hamsters but no SARS‐CoV‐2‐typical vascular or bronchial lesions in OTS‐300‐immunized hamsters. Bar 100 µm, from left to right: mock, mRNA, OTS‐300. (i, j) Antigen detection: Samples were screened for SARS‐CoV N‐protein. (i) Antigen score with median and interquartile range and (j) representative immunohistochemistry lung sections illustrate antigen in both pneumocytes (green arrow) and bronchial epithelial cells (green arrowhead) in nonimmunized control hamsters, the multifocal presence of antigen only in pneumocytes (green arrow) in hamsters immunized with mRNA, and the absence of viral antigen in OTS‐300‐immunized hamsters. Bar 100 µm, from left to right: mock, mRNA, OTS‐300. *p < 0.05, **p < 0.01, ***p < 0.001, ****p < 0.0001. (d) Mixed‐effects model with Geisser–Greenhouse correction, group comparison with Tukey's multiple comparisons test. (e) Two‐way ANOVA, group comparisons with Tukey's multiple comparisons test. (g, i) Kruskal–Wallis test followed by Dunn's multiple comparisons test.

We utilized the well‐evaluated and safe OTS‐228 SARS‐CoV‐2 LAV [10, 15] as a backbone and modified the sequence encoding for the spike protein. Attenuation of OTS‐228 was achieved by the “one‐to‐stop” codon modification of serine and leucine codons in the ORF1ab introduced by yeast transformation‐associated recombination (TAR) cloning. Attenuation is further facilitated by a combination of point mutations (K164A and H165A) in the nonstructural protein 1 gene and deletions of ORF6‐8, as well as the spike polybasic cleavage site (PCS) (Figure 1a). This strategy allowed the use of OTS‐228 as a platform to express spikes of different virus variants, as shown here by exchanging the ancestral SARS‐CoV‐2 spike protein coding sequence with the spike coding sequence of XBB.1.5 (EPI_ISL_15312903). The new OTS construct was designated as “OTS‐300” (Figure 1a).

### 2.2 OTS‐300 Remains Attenuated and Nontransmissible in Syrian Hamster Model

To confirm that the attenuated phenotype is retained with the updated spike sequence, we intranasally inoculated serologically naïve Syrian hamsters with OTS‐300 (10^4^ TCID_50_/hamster) and monitored body weight changes and SARS‐CoV‐2 genome loads in nasal wash samples (Figure 1b). Comparisons were made against hamsters inoculated with noninfectious cell culture medium (mock) or i.m. vaccinated with one dose of mRNA vaccine encoding the XBB.1.5 spike protein. None of the groups exhibited body weight loss following vaccination (Figure 1c; Figure S1a). To assess potential unintended shedding of OTS‐300, we co‐housed six serologically naïve contact animals in a 1:1 setup, forming six transmission pairs per group, beginning one day postvaccination (dpv) (Figure 1b). None of the contact animals showed any body weight loss irrespective of the experimental group (Figure S1b,c).

OTS‐300 genomic RNA was detected in nasal wash samples at 3 dpv in all vaccinated animals with a median of 10^6.46^ genome copies per mL (gc/mL) and decreased 7 dpv (median 10^3.59^ gc/mL) (Figure S1d). While the vaccine virus genome was still detectable in 10 out of 16 animals at 7 dpv, it was only detectable in a single residual positive animal at 19 dpv (Figure S1d). No transmission of the vaccine virus occurred as demonstrated by the absence of viral genome in the nasal wash samples of contact animals (Figure S1e), which all remained serologically naïve (Figure S1f).

### 2.3 OTS‐300 Efficiently Protects From Disease

To assess the quality of vaccine‐induced protection, we challenged animals with SARS‐CoV‐2 XBB.1.5 at 21 dpv, comparing outcomes across the OTS‐300, mRNA, and mock vaccinated groups. The XBB.1.5 challenge caused significant body weight loss in mock‐immunized animals, but no mortality or severe clinical disease in any of the groups. One animal of the mock group exhibited a bloody nasal washing sample at 5 dpc and did not regain consciousness following short‐term anesthesia, but it remained unclear whether this was infection‐related (Figure S2a). Significant differences in relative body weight changes were observed in mock compared with mRNA or OTS‐300 immunized animals (Figure 1d; Figure S2b). While the mock‐vaccinated group lost 6.2% of their initial body weight on average by day 5 postchallenge, animals in the OTS‐300 group gained body weight, and the mRNA group maintained stable body weight over the first 5 days postchallenge (Figure 1d; Figure S2b).

Infectious viral loads in organs were titrated by endpoint dilution assay. By 2 dpc, infectious viral loads could be detected in nose and lung samples of mock (median TCID_50_> 10^3^) and mRNA (median TCID_50_> 10^2^) immunized animals. In contrast, no infectious viral particles were detected in the nose or lung of OTS‐300 immunized animals (Figure 1e; Figure S2c). This was in line with the analysis of viral genome loads in respiratory tissues with RdRp gene‐specific RT‐qPCR, which showed that the OTS‐300 group had minimal (<10^5^ gc/mL) or no detectable viral genome loads in the lower respiratory tract (LRT) 2 dpc, while the mock group exhibited the highest viral loads (median of 4.2 × 10^7^ gc/mL) and the mRNA group (median of 3.6 × 10^6^ gc/mL) displayed intermediate levels (Figure S2d). Five days post‐challenge, infectious viral particles only remained detectable in the lungs of one mock and one mRNA immunized animal (Figure S2c). At the same time point, OTS‐300‐vaccinated animals only showed minimal numbers of viral genome in the nose, but were tested completely negative for viral RNA in the lungs, while all mock and the majority of mRNA immunized animals still tested positive in the LRT (Figure S2e). By day 14 postchallenge, only trace amounts of genome copies per mL (<10^5^) were detected, primarily in conchae samples of the mock and mRNA groups (Figure S2f). Notably, one mock‐vaccinated animal that failed to regain consciousness after brief anesthesia at 5 dpc showed high viral loads (>10^7^ gc/mL) across lung tissues, indicating that challenge infection could have contributed to this outcome (Figure S2e).

The histopathological examination of the lungs 5 dpc revealed that OTS‐300‐vaccinated hamsters showed no evidence of SARS‐CoV‐2 challenge infection and related pneumonia‐induced atelectasis (Figure 1f–h), confirmed by the absence of viral antigen (Figure 1i,j). Only a slight expansion of the interstitium by immune cell infiltration, predominantly lymphocytes, was observed in the majority of animals, consistent with an immunostimulation that occurred some time ago. In contrast, all mRNA‐vaccinated animals exhibited peribronchial, perivascular, and interstitial infiltration by macrophages, some heterophilic granulocytes, and lymphocytes in the lungs (Figure 1h). Three mRNA‐immunized hamsters showed pulmonary atelectasis (Figure 1f,g), two of them with mild vasculitis and one with necrotizing bronchitis. Focal to oligofocal sparse lesion‐associated antigen was observed in all hamsters (Figure 1i,j). These findings are consistent with an acute SARS‐CoV‐2 infection. All five mock‐immunized hamsters showed pulmonary atelectasis and acute SARS‐CoV‐2 characteristic vascular lesions, necrotizing bronchitis, and marked alveolar edema with mixed immune cell infiltration (Figure 1f–h). Intralesional, multifocal to coalescing viral antigen was detected in all mock‐immunized hamsters (Figure 1i,j).

### 2.4 OTS‐300 Vaccination Efficiently Reduces Challenge Virus Transmission Compared with Other Treatment Groups

**FIGURE 2:**
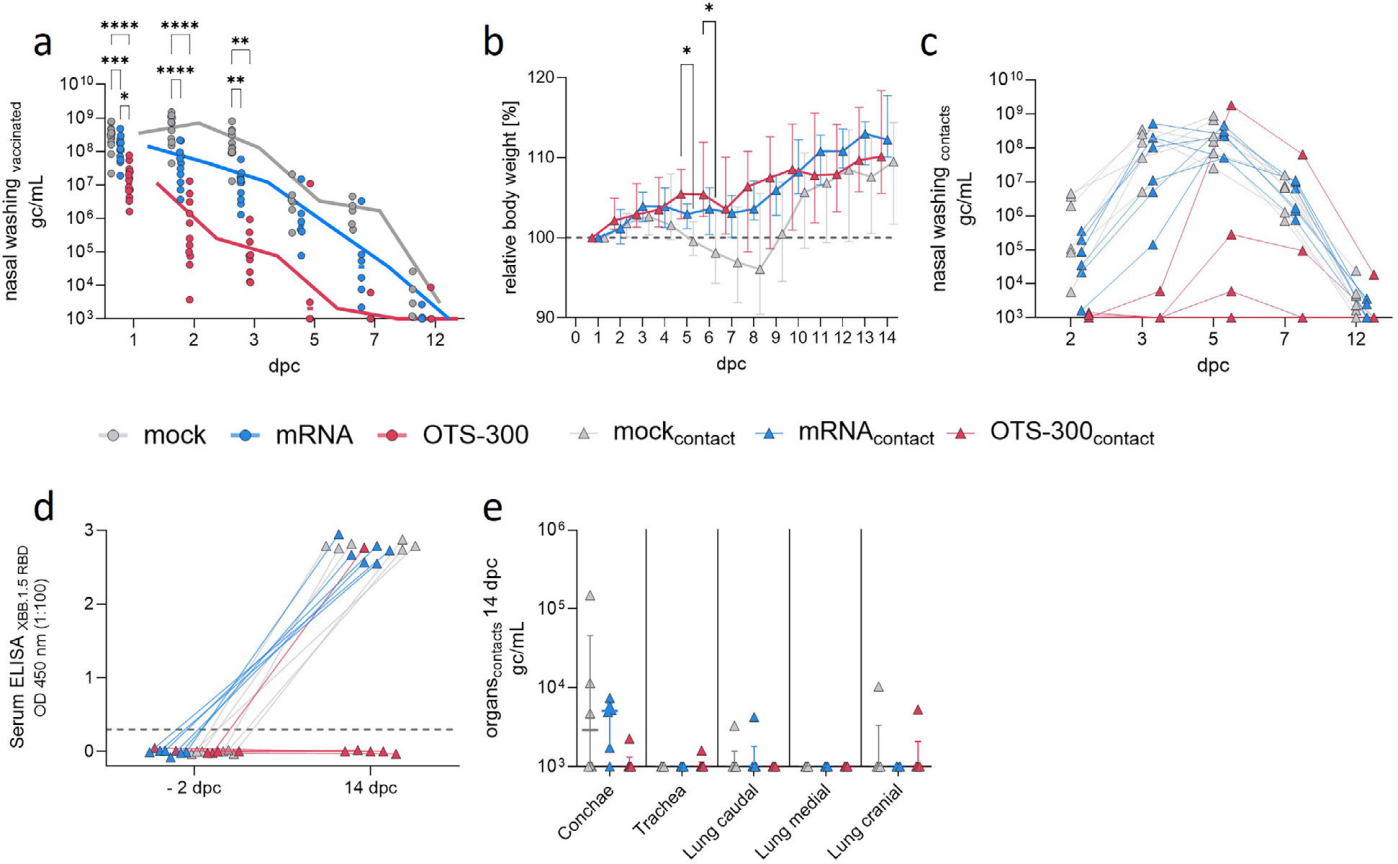
OTS‐300 vaccination reduces SARS‐CoV‐2 XBB.1.5 shedding and transmission. (a) SARS‐CoV‐2 genome copies per mL (gc/mL) in longitudinal nasal wash samples by RT‐qPCR in vaccinated animals. Lines represent median gc/mL with *n* = 16 for dpc1, *n* = 11 for dpc 2–3, *n* = 6 for dpc 5–12. Two‐way ANOVA with Geisser–Greenhouse correction, group comparisons with Tukey's multiple comparisons test. (b) Median relative body weight with interquartile range of contact animals. Two‐way ANOVA with Geisser–Greenhouse correction, group comparison with Dunnett's multiple comparisons test. (a, b) *p < 0.05, **p < 0.01, ***p < 0.001, ****p < 0.0001. (c) SARS‐CoV‐2 genome copies per mL (gc/mL) in longitudinal nasal wash samples by RT‐qPCR in contact animals, *n* = 6. (d) SARS‐CoV‐2 XBB.1.5 RBD‐specific serum antibodies in contact animals at two days before challenge (−2 dpc) and dpc14 detected with multispecies ELISA at 1:100 dilution. (e) SARS‐CoV‐2 genome copies per mL (gc/mL) with median and interquartile range in organs analyzed by RdRp gene‐specific RT‐qPCR 14 dpc.

In order to evaluate shedding of the challenge virus, we analyzed viral genome copy numbers in longitudinal nasal wash samples. Varying levels of viral shedding were observed, depending on the vaccine administered. Following the first 3 days postchallenge, viral loads were significantly reduced in mRNA and OTS‐300‐vaccinated animals compared with the mock group (Figure 2a). However, OTS‐300‐vaccinated animals also showed significantly lower viral genome loads compared with the mRNA group (Figure 2a). While viral genome copies were still detectable in all mRNA‐vaccinated animals at 7 dpc, they were already below the limit of detection in the majority of OTS‐300 vaccinated animals at that timepoint (Figure 2a).

None of the serologically naïve contact animals, which were co‐housed in a 1:1 setup with the challenged animals at 1 dpc (six transmission pairs per group), reached the humane endpoint during the 13‐day contact period (Figure S2g). Contact animals in the mock group showed body weight loss between 5 and 9 dpc (4–8 days post‐co‐housing) (Figure 2b), in contrast to those in contact with the mRNA or OTS‐300 vaccinated animals, except for one OTS‐300 contact animal (Figure S2h). Weight loss at 5 and 6 dpc was significantly higher in contact animals co‐housed with the mock group compared with the OTS‐300 group (Figure 2b). Nasal wash samples and RBD ELISA showed that all contact animals in the mock and mRNA groups acquired SARS‐CoV‐2 and showed similar shedding values of up to 10^8^ gc/mL four days post co‐housing (Figure 2c,d) whereas viral genome copies were only detected in three out of six of the contact animals of the OTS‐300 group with one animal displaying a very low viral genome load (Figure 2c). Interestingly, only one contact animal of the OTS‐300 group reached shedding values above 10^7^ gc/mL and seroconverted (Figure 2c,d). In contrast, all mRNA and mock contact animals seroconverted. Notably, while virus transmission to contact animals of the mRNA and mock group already occurred after 1 day of co‐housing, it was delayed to 2–4 days after co‐housing in OTS‐300 contact animals (Figure 2c). Efficient infection with seroconversion did not occur in those animals that only showed low levels of detectable virus genome copies after four days of co‐housing. Examination of tissue samples from contact animals at 14 dpc indicated viral genome presence predominantly in the nose, with only minor amounts in LRT samples for all groups. One single animal of the OTS‐300 group that seroconverted had low detectable amounts of viral genome copies in LRT tissue samples (Figure 2e).

### 2.5 OTS‐300 Mediates Fast and Robust Mucosal and Systemic Antibody Response

**FIGURE 3:**
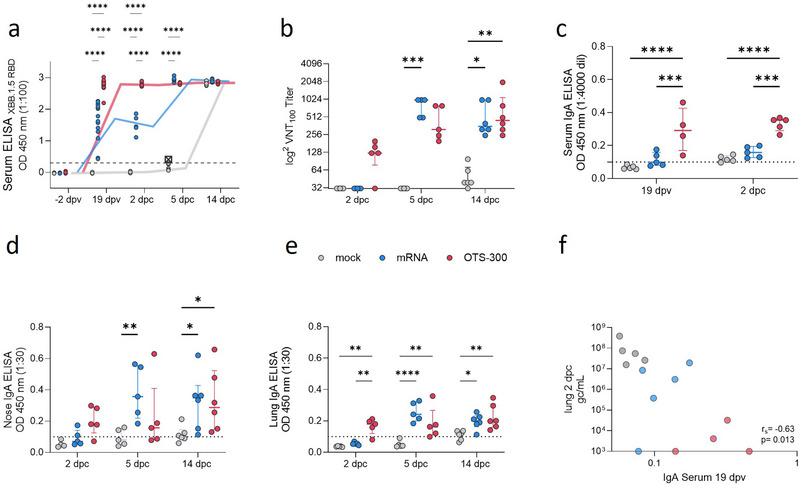
OTS‐300 vaccination elicits a fast systemic and mucosal immune response. (a) Longitudinal SARS‐CoV‐2 XBB.1.5 RBD‐specific serum antibodies at −2 dpv (*n* = 16), 19 dpv (*n* = 16), 2 dpc (*n* = 5), and 14 dpc (*n* = 6) detected with multispecies ELISA at 1:100 dilution. Lines represent median OD_450 nm_. Crossed symbol: died during narcosis 5 dpc. (b) Individual and median serum neutralization titer assessed in a live virus neutralization test with SARS‐CoV‐2 XBB.1.5 (capacity to neutralize 100 TCID_50_). (c) IgA levels in serum, (d) nose, and (e) lung were analyzed using an XBB.1.5 Spike‐specific IgA ELISA at (c) 1:4000 or (d, e) 1:30 dilution and displayed as the OD 450 nm. *p < 0.05, **p < 0.01, ***p < 0.001, ****p < 0.0001. (b–e) Two‐way ANOVA followed by Tukey's multiple comparisons test. (f) Spearman correlation test of average viral loads in the lung (average of cranial, medial, and caudal) and Spike‐specific Serum IgA 19 dpv.

We aimed to characterize mucosal and systemic immune responses to vaccination and challenge infection in Syrian hamsters. Humoral responses against the receptor‐binding domain (RBD) are considered protective against SARS‐CoV‐2 [23, 26]. Therefore, serum samples were analyzed for antibodies against SARS‐CoV‐2 using an RBD‐based multispecies ELISA [27], based on the Omicron XBB.1.5 variant [28]. Antibody levels in serum at 19 dpv were significantly higher in OTS‐300 compared with mRNA vaccinated animals (Figure 3a). Following the challenge infection with XBB.1.5, serum antibodies in the OTS‐300 group remained significantly higher compared with the mRNA and the mock group at 2 dpc. By contrast, it took the mRNA group until 5 dpc to reach comparable antibody levels (Figure 3a). The mock‐vaccinated animals seroconverted later, showing no detectable antibodies at 5 dpc, but reaching maximum OD by 14 dpc (Figure 3a).

To assess the neutralizing capacity of the antibodies, we tested postchallenge samples using a surrogate virus neutralization test, in fact, testing for the functional block of RBD‐ACE2 binding. Two days after challenge infection, the OTS‐300 group reached nearly 100% neutralizing capacity, while the mRNA group showed a mean neutralizing capacity of 59%, and the mock group exhibited a low mean of 4.5% (Figure S3a). By 5 dpc, the mRNA group also reached a maximum neutralizing capacity of 100%, and the mock group increased to 58.7% in this surrogate virus neutralization test (Figure S3a).

To validate these ELISA‐based findings, we assessed live virus neutralization capacity against SARS‐CoV‐2 Omicron XBB.1.5. Using an in vitro cytopathic effect (CPE)‐based virus neutralization test (VNT), we determined the maximal serum dilution capable of fully neutralizing 100 TCID_50_ (VNT_100_) (Figure 3b). At 2 dpc, the mock and mRNA sera were below the threshold of VNT_100_<32, while four out of five OTS‐300 samples tested positive, with a median VNT_100_ titer of 124 (Figure 3b). By 5 dpc, all mRNA and OTS‐300 samples tested positive, with median VNT_100_ titers of 992 and 312, respectively, while mock samples remained below the threshold (Figure 3b). Two weeks postchallenge, mRNA and OTS‐300 samples continuously exhibited high VNT_100_ titers (median VNT_100_ titers of 353 and 445), and five of six mock animals also displayed some neutralizing capacity (median VNT_100_ titer of 39) (Figure 3b).

To quantify serum and mucosal IgA antibodies, we performed a SARS‐CoV‐2 Omicron XBB.1.5 spike‐specific IgA ELISA using serum (Figure 3c) as well as supernatants of homogenized nose (Figure 3d) and lung (Figure 3e) samples. Already 19 dpv and 2 dpc, we detected significantly higher serum IgA levels in OTS‐300 vaccinated animals compared with the mRNA group (Figure 3c). Furthermore, at 2 dpc, OTS‐300 immunized animals displayed significantly higher levels of total XBB.1.5 specific Ig in nasal samples compared with mock and mRNA immunized animals (Figure S3b), and four out of five OTS‐300 vaccinated animals compared with two out of five mRNA vaccinated animals showed detectable levels of IgA (Figure 3d). Similarly, OTS‐300 immunized animals had significantly higher levels of IgA (Figure 3e) and total XBB.1.5 specific Ig (Figure S3c) compared with mock and mRNA immunized animals in lung samples at 2dpc. By 5 dpc, all vaccinated animals showed robust IgA levels in the lung, however in the mock group, mucosal IgA could only be detected 14 dpc (Figure 3e). Serum IgA levels 19 dpv correlated negatively with viral loads detected in the lungs 2 dpc, suggesting a protective role (Figure 3f). Additionally, IgA but not total Ig levels and viral loads in nasal samples 2 dpc correlated negatively (Figure S3d,e). Even if not statistically significant, there was a trend to increasing neutralizing activity of homogenized nasal tissues from mock (1.1/0.7% inhibition (mean/median) to mRNA (3.3/1.4% inhibition (mean/median) to OTS‐300 (17.5/12% inhibition (mean/median) measured by SARS‐CoV‐2 neutralizing antibody ELISA (Figure S3f). Thus, OTS‐300 induced mucosal and systemic antibody responses, which were accelerated compared with mRNA vaccination and contributed to protection against XBB.1.5.

In contrast to neutralizing antibody binding epitopes in the spike protein, T cell epitopes appear relatively conserved between SARS‐CoV‐2 variants [3, 29, 30]. Previous studies indicated protective functions of memory T cells against SARS‐CoV‐2 in Syrian hamsters [17, 21], but tools for the detection of SARS‐CoV‐2‐specific T cells by flow cytometry had not yet been established in this model. Thus, we established a proliferation assay to detect SARS‐CoV‐2 spike‐specific CD4 and CD8 T cells. Spike peptide pool reactive CD4 and CD8 T cells were detected in all groups 14 dpc, except for a naïve contact animal without significant differences (Figure S4a,b). Albeit not statistically significant, OTS‐300‐vaccinated animals showed the highest percentage of CD8 Spike‐specific T cells 14 dpc (Figure S4c). In summary, both CD4‐ and CD8‐specific T cells could be detected regardless of the vaccination group.

### 2.6 Decreased Inflammatory Responses Correlate With Lower Viral RNA Loads in OTS‐300 Vaccinated Animals

**FIGURE 4:**
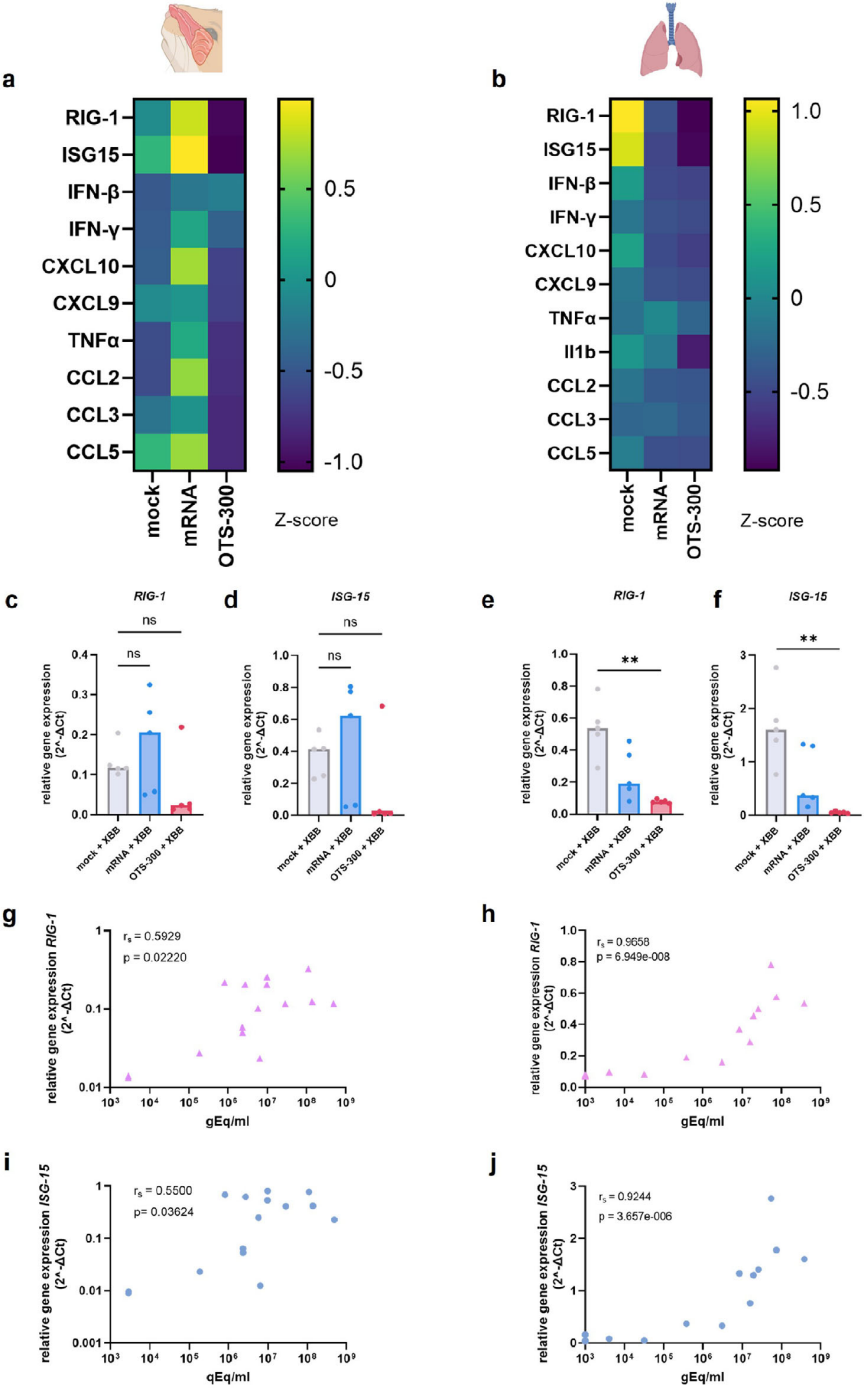
Reduced viral RNA loads in OTS‐300 vaccinated animals correlate with decreased inflammation in the lung and nose 2 dpc. Relative gene expression of proinflammatory and early cytokines 2 dpc characterized by qPCR (*n* = 5). (a, b) heatmap of median Z‐scored normalized relative gene expression in (a) nose and (b) lung. Individual and median relative gene expression are displayed for (c, e) *RIG‐1* and (d, f) *ISG‐15* in (c, d) nose and (e, f) lung. (g–j) Spearman correlation (*r*_s_) of the relative gene expression 2 dpc and average viral genome copies in (g, i) nose and (h, j) lung (cranial, medial, and caudal). *p* < 0.05, *p* < 0.01. (c–f) Kruskal–Wallis test followed by Dunn's multiple comparisons test.

Since we observed reduced viral RNA loads, indicating only minimal challenge virus replication in OTS‐300‐vaccinated animals, we expected reduced virus‐induced mucosal inflammatory responses. Thus, we characterized the innate inflammatory response in the nose and lung 2 and 5 dpc by qPCR (Figure 4; Figures S5 and S6). Overall, OTS‐300 vaccinated animals showed reduced transcription of genes coding for pro‐inflammatory cytokines, chemokines, interferons, and interferon‐inducible genes in the nose and lung when compared with mock and mRNA vaccinated animals (Figure 4a,b; Figures S5 and S6). For example, 2dpc, mock‐vaccinated animals showed a trend toward upregulation of *RIG‐I*, *ISG‐15*, and *CXCL‐10* in the nose (Figure 4c,d) and significantly upregulated expression in the lungs compared with OTS‐300‐vaccinated animals (Figure 4e,f; Figure S5a). Chemokines associated with monocyte and T cell attraction, *CCL‐2* and *CCL‐5*, were also significantly upregulated in the mock animals compared with the OTS‐300 group. In both the nose and lung, the relative gene expression of *RIG‐I* and *ISG‐15* at 2 dpc was positively correlated with the viral load measured by RT‐qPCR (Figure 4g,h). In conclusion, the OTS‐300 vaccination led to constrained virus‐induced mucosal inflammatory responses following challenge infection, correlating with reduced viral titers.

## 3 Discussion

LAVs represent a promising prophylactic tool against SARS‐CoV‐2 infection, especially if they are adaptable to target VOCs. In future vaccination strategies, LAVs could play a pivotal role. Technologies, such as reverse genetics and codon deoptimization, have accelerated the development of safe LAV, which may be used as versatile vaccine platforms, also allowing rapid antigen updates. As shown by our previous work, OTS‐modified LAV based on the ancestral SARS‐CoV‐2 proved to be safe and effective against SARS‐CoV‐2 challenge infection in preclinical models [10, 15]. Vaccination with OTS‐228, carrying the ancestral spike protein, induced sterilizing immunity against homologous challenge infection with WT SARS‐CoV‐2 and broad protection against Omicron BA.2, BA.5, and XBB.1.5. Although OTS‐228 vaccinated animals showed reduced XBB.1.5 shedding, complete prevention of transmission to contacts was not achieved. This highlights the need for an adaptable LAV that can be antigenically matched to drifted SARS‐CoV‐2 variants and maintain their transmission‐blocking capacity [10].

Here, we characterized the safety and efficacy of the XBB.1.5 spike updated candidate OTS‐228, designated as OTS‐300. Beyond antibody escape, spike glycoprotein mutations can affect viral traits like ACE2 binding, spike stability, fusogenicity, and overall fitness [1, 31, 32, 33]. Unlike vector‐based mucosal vaccines [7, 34], the OTS vaccine is based on an attenuated ancestral SARS‐CoV‐2, enabling it to mimic natural infection and fully engage the immune system. In theory, introducing spike mutations into OTS‐300 could also alter LAV characteristics.

However, the adapted vaccine candidate was well tolerated, did not induce weight loss in the vaccinated animals, and was not transmitted to contact animals. Therefore, the adapted spike protein did not alter the safety and attenuation of the vaccine. This robust attenuated phenotype of the OTS LAVs was achieved via large‐scale OTS‐codon modifications in combination with additional attenuating mutations and deletions [10, 15]. To reduce the number of animals with respect to the 3R principle, no side‐by‐side comparison of OTS‐228 and OTS‐300 was performed. Performance of OTS‐228 against XBB.1.5 challenge infection was described in previous studies [10]. Our findings underscore the adaptability of the OTS vaccine platform, thereby establishing it as a valuable tool against emerging SARS‐CoV‐2 variants.

Both mRNA and OTS‐300 protected hamsters against weight loss after XBB.1.5 challenge infection and reduced viral RNA loads in the LRT. However, OTS‐300 protected from virus propagation into the LRT more efficiently compared with one dose of mRNA vaccination, as shown by the complete absence of infectious viral particles in the nose and lung 2 dpc. We recognize that the recommended vaccination regimen for SARS‐CoV‐2 mRNA vaccination in humans is a prime‐boost regimen [31]. Booster vaccination could potentially have improved mRNA vaccine efficacy. However, we wanted to assess the protective capacity and immune responses after single‐shot prime vaccination with OTS‐300. Our studies show that even a single‐shot immunization with OTS‐300 induced a robust protective immune response. Although low levels of viral genome copies were found in nasal washings of animals co‐housed with OTS‐300‐immunized animals after challenge infection, only one out of six seroconverted by 14 dpc. On the contrary, all six contact animals in both the mRNA and mock groups seroconverted. Single‐shot OTS‐300 immunization reduced transmission of SARS‐CoV‐2 XBB.1.5 to contact animals, which correlated with reduced infectious viral loads in the upper respiratory tract. Our findings are in line with other studies, which confirmed that intramuscular vaccination, for example, with mRNA or inactivated vaccines mainly reduces viral loads in the LRT, but not efficiently in the upper respiratory tract [7, 32, 33]. In contrast, intranasal vaccination with other LAV efficiently reduced viral loads in the upper respiratory tract and consequently shedding, following challenge infection [14, 22].

Neutralizing humoral antibodies, a well‐known correlate of protection against COVID‐19, were detected instantly after challenge infection in the OTS‐300 group, but with a delay in the mRNA‐immunized animals. Yet, to effectively induce sterilizing immunity against respiratory viruses, local immunity must be established and should therefore be targeted by vaccination approaches to reduce virus transmission. Human challenge studies identified early mucosal IgA responses as a correlate of protection against SARS‐CoV‐2, as they are strongly associated with virus clearance [23]. Along with other studies [7, 34, 35], this implies that vaccines should be designed to optimally target mucosal IgA responses to reduce transmission. Whether such mucosal IgA can be induced by parenteral vaccination independent of infection remains unclear [34, 35, 36]. Recent studies indicate that repeated mRNA vaccination boosts mucosal IgG but fails to effectively induce mucosal IgA [6, 9, 37, 38]. Other studies suggest that repeated mRNA vaccination may enhance a temporary mucosal IgA response, but it necessitates high serum antibody levels, and prior infection appears to increase the ability to boost mucosal IgA [39]. Further, mucosal IgA can be boosted efficiently in systemically mRNA primed animals, for example, by intranasal protein vaccination [40]. Here, mucosal IgA was detected promptly after challenge in nasal and lung samples of the OTS‐300‐vaccinated group, while these responses were delayed in the mRNA and mock groups. These accelerated mucosal antibody responses in OTS‐300‐immunized animals are associated with higher IgA and nAb titers in serum at the timepoint of challenge infection. Notably, despite the absence of infectious virus in nasal samples of OTS‐300‐immunized animals, nAb levels in nasal samples at 2 dpc remained below the limit of detection in most animals, indicating limited sensitivity of established neutralization assays for mucosal samples. Nevertheless, there was a nonsignificant trend toward increasing nAb levels in OTS‐300‐immunized animals. Importantly, the rapid viral clearance observed in the OTS‐300 vaccinated group correlated with elevated serum IgA levels at 19 dpv and increased nasal IgA but not total XBB.1.5 binding Ig at 2 dpc, and led to a reduction in virus‐induced innate inflammatory responses in the respiratory tract.

Comprehensive understanding of origin and production of antibodies at mucosal sites, for example, roles of active transport or transudation, requires further investigations [6, 9, 35, 37, 38, 41]. While serum exudation or transport of systemic antibodies to the mucosa appears limited but possible, the majority of mucosal antibodies, especially IgA, likely originate from tissue resident lymphocytes [35, 38]. The establishment of tissue resident memory B cells (BRMs) in the lung requires local antigen display [7, 35, 42]. In contrast to circulating B cells in lymphoid organs, BRMs do not enter circulation and remain in the respiratory tract. Upon secondary antigen encounter, they can quickly differentiate into antibody‐secreting cells [42]. It has been reported that the response time of tissue‐resident BRMs is faster than that of circulating B memory cells, which might be due to the close location at the site of viral entry or qualitative differences [35, 42]. Although we did not directly analyze B cell responses, we assume that intranasal vaccination with OTS likely induced lung‐resident BRMs, which contributed to the accelerated antibody response at the mucosal sites that we detected.

Substantial mutations in the spike protein enable VOCs to escape from neutralizing Abs and B cell recognition, whereas T cell epitopes appear to be more conserved, facilitating cross‐recognition [3, 29, 30, 43, 44]. In mice, we showed previously that OTS‐228 induces SARS‐CoV‐2‐specific memory T cells in lungs [10]. While numerous studies suggest that T cells play a protective role against SARS‐CoV‐2, their function in Syrian hamsters has yet to be fully determined [17, 20, 21]. To our knowledge, methods for the detection of SARS‐CoV‐2‐specific CD8 and CD4 T cells in this animal model had not been established so far. We therefore established a proliferation assay to detect SARS‐CoV‐2‐specific T‐cells in Syrian Hamsters using flow cytometry. We did not observe statistically significant differences in SARS‐CoV‐2‐specific T cell responses between vaccinated and mock‐immunized animals 14 dpc in the lungs. However, we noticed a trend toward a strong SARS‐CoV‐2 spike‐specific CD8 T cell response in OTS‐300‐vaccinated animals. Such a CD8 T cell response at the initial site of viral infection could be another benefit of mucosal vaccination, contributing to fast viral clearance and cross‐protection against new variants [3, 23, 24, 29, 30, 43, 44]. As an attempt to reduce the number of animals in this study, following the 3R principle, we did not sacrifice animals before the challenge infection. Therefore, no data regarding the T cell response in the absence of the challenge infection is available. Nevertheless, the results suggest that despite the broad absence of viral antigen in the lung in the majority of OTS‐300‐vaccinated animals after challenge infection, a robust T cell response was either maintained after vaccination or elicited after challenge. The assay established in this study will allow the analysis of SARS‐CoV‐2‐specific T cell responses in Syrian hamsters in future investigations. However, a more comprehensive understanding of the cellular immune response to intranasal LAV vaccination in this highly relevant animal model for respiratory virus infections will require further research [17, 18, 45].

In summary, we demonstrated that the OTS LAV platform can be easily adapted to emerging VOCs through spike protein exchange or modification. The safety profile and the substantial attenuation observed with OTS‐228 were consistently maintained with OTS‐300. Importantly, intranasal vaccination with OTS‐300 efficiently protected Syrian hamsters against SARS‐CoV‐2 XBB1.5 replication in the lungs, as well as disease, inflammation, and transmission, likely due to the development of potent systemic and rapid mucosal antibody responses. Our findings position OTS‐300 and the OTS platform technology as a promising, adaptable vaccination tool, with the potential to significantly reduce breakthrough infections and transmission, thereby helping to curb the emergence of new viral strains.

### 3.1 Data Limitations and Perspectives

To reduce animal use in line with the 3R principle, animals were not sacrificed before challenge infection, limiting the availability of baseline T cell response data. Despite this, our findings suggest that OTS‐300 vaccination either maintained or elicited a strong T cell response after challenge, even in the absence of detectable viral antigen in the majority of lungs. The assay established here provides a valuable tool for characterizing SARS‐CoV‐2–specific T cell responses in Syrian hamsters. Future studies are needed to achieve a more comprehensive understanding of the cellular immune response to intranasal LAV vaccination in this model.

## 4 Methods

### 4.1 Animals

Specific pathogen‐free Syrian hamsters (*Mesocricetus auratus*), with an equal number of males and females, were obtained from Janvier Labs (www.janvier‐labs.com) at an age of 5 weeks.

### 4.2 Sampling and Processing

To quantify virus shedding, each nostril was washed with 200 µL PBS using a micropipette, collecting the reflux in a safe lock sample tube under isoflurane anesthesia for less than a minute. Body weight was determined by putting the hamsters on a fine scale with a *d*‐value of 0.1 g. Physical well‐being was assessed on a daily basis by qualified personnel through visual inspection. Body weight loss of equal or more than 20% postinoculation was defined as the human endpoint. At distinct timepoints, animals were euthanized, and upper respiratory (conchae nasalis and trachea) and lower respiratory tract samples (lung cranial, medial, and caudal) of about 0.1 cm^3^ were taken to examine viral genome loads in these organ samples. Organ samples were homogenized in a 1 mL mixture composed of equal volumes of Hank's balanced salts MEM and Earle's balanced salts MEM with 1% penicillin–streptomycin at 300 Hz for 2 min using a Tissuelyser II (Qiagen) and were then centrifuged to clarify the supernatant. Besides blood collection at the end time points, serological follow‐up samples were taken by puncture of the saphenous vein under isoflurane anesthesia. Serum, nasal washes, and organ samples were stored at −80°C.

### 4.3 Hamster Vaccination

Three groups of hamsters of 16 animals each were vaccinated by applying either intranasally 70 µL (35 µL per nostril) of OTS‐300 (10^4.2^ TCID_50_/hamster) or the mRNA‐vaccine (5 µg Raxtozinameran (Comirnaty Omicron XBB.1.5) in 50 µL volume/hamster) intramuscularly. The mock group was intranasally inoculated with 70 µL (35 µL per nostril) of noninfectious cell culture medium. Six serologically naïve direct contact animals per group were included in the study. Six of the vaccinated animals were paired with the six contact animals, forming six transmission pairs per group. The contact animals were introduced one day after vaccination to evaluate unintended vaccine transmission. To evaluate vaccine shedding and replication in the nasal cavity, nasal shedding samples were taken two days before vaccination, and 3, 7, and 19 dpv. Body weight was tracked daily over the first 7 dpv. Serum samples were obtained before challenge at −2 dpc, which correlates to 19 dpv.

### 4.4 Hamster Challenge Infection

At 21 dpv, the inoculated animals of the mock, mRNA, and OTS‐300 group were intranasally infected with 70 µL SARS‐CoV‐2 XBB.1.5 hCoV‐19/Netherlands/NH‐EMC‐5667/2023 (EPI_ISL_16640568) (second passage after propagation on Vero E6, TCID50 10^4.47^/hamster), and direct contact animals were separated for 24 h postchallenge infection to prevent unspecific transmission of the inoculum. To detect virus shedding, nasal washing samples of inoculated and contact animals were taken at 1, 2, 3, 5, 7, 12, and 14 dpc. Virus replication in the respective upper and lower respiratory tissues was evaluated following euthanasia of five inoculated hamsters per group at 2 and 5 dpc, as well as the six transmission pairs at 14 dpc. Serum samples were obtained at the time point of euthanasia from the respective animals.

### 4.5 Quantification of Infectious Viral Particles

Infectious viral particle titres were quantified using tissue culture infectious dose 50%. Vero E6 cells were seeded in 96‐well plates one day before titration and inoculated with a 10‐fold serial dilution of the samples starting at 10^−1^. Two plates with eight replicates each were used per sample. Following inoculation, plates were incubated at 37°C in a humidified atmosphere with 5% CO_2_ for three days. The cells were then examined microscopically for the presence of virus‐induced CPE. The dilution at which 50% of the wells showed CPE corresponds to the tissue culture infectious dose 50 (TCID_50_), as determined by the endpoint dilution method. The TCID_50_ was calculated according to the Spearman–Kaerber formula per plate. Final titre was defined as the mean of both plates.

### 4.6 RNA Extraction and RT‐qPCR for Detection of Viral RNA

Nucleic acids were extracted from 100 µL of nasal wash or organ sample supernatant using the NucleoMag Vet kit (Macherey Nagel). SARS‐CoV‐2 detection in the RdRp gene was carried out with primers and probes recommended by the Pasteur Institute: SARS2‐IP4‐14059F (forward), SARS2‐IP4‐14146R (reverse), and probe SARS2‐IP4‐14084 [46]. Beta‐actin was detected as a control for RNA extraction using specific primers [47]. The RT‐qPCR was set up with the qScript XLT One‐Step RT‐qPCR ToughMix (QuantaBio) in a 12.5 µL reaction, including 1 µL of the FAM mix and 2.5 µL of RNA. The protocol involved 10 min at 50°C for reverse transcription, 1 min at 95°C for activation, and 42 cycles of denaturation (95°C), annealing (60°C), and elongation (68°C), with fluorescence measured during annealing. RT‐qPCR was performed on a BioRad CFX96 system, and a standard dilution was used to calculate viral genome copies per mL.

### 4.7 RNA Extraction and qPCR for Detection of Cytokine Expression

Organs were homogenized in 1 mL Trizol using a Miltenyi GentleMACS tissue dissociator with the RNA program 1. Samples were stored at −80°C before RNA isolation. To isolate RNA, 200 mL RNAse‐free Chloroform was added to 1 mL homogenate in Trizol and mixed. Following 3 min incubation at RT, samples were centrifuged for 15 min at 12,000×*g* at 4°C. The aqueous phase was transferred into new 1.5 mL tubes. After adding 20 µL glycogen (Thermo Fisher Scientific) and 200 µL precooled isopropanol, the samples were incubated at −20°C for 60 min. Samples were then centrifuged for 15 min at 12 000×*g* at 4°C to precipitate RNA. Supernatant was removed, and samples were washed twice with 400 µL 75% RNAse free EtOH. Pellets were solubilized in 20 µL H_2_O at 60°C for 15 min. RNA isolated from samples 5dpc was processed as described for the detection of viral RNA.

RNA was transcribed into cDNA using the High‐Capacity cDNA reverse transcription kit (Thermo Fisher Scientific) following the manufacturer's recommendations. For the qPCR, primers (Table S1) were used at 0.25 µM with 5 µL PowerUp SYBR Green Mastermix (Thermo Fisher, Applied Biosystems) and 2 µL cDNA in a total reaction volume of 10 µL. Detailed protocol is provided by the manufacturer (https://assets.thermofisher.com/TFS‐Assets/LSG/manuals/MAN0013511_PowerUp_mastermix_UG.pdf; MAN0013511 Revision F.0). The qPCR was performed using a Quant Studio 6 Flex Real‐time PCR cycler (Thermo Fisher Scientific, Applied Biosystems). The PCR conditions were set as described for the standard cycling mode in the manufacturer's guidelines, starting with UDG activation at 50°C for 2 min, followed by the initial denaturation at 95°C for 2 min, then cycle denaturation at 95°C for 15 s and an extension at 60°C for 30 s for 40 cycles. For the melt curve analysis, we set 95°C for 15 s, then 60°C for 1 min, followed by 95°C for 15 s. Samples were tested in duplicates. Relative gene expression was determined as 2^−ΔCt^, where ΔCt is the difference in average threshold cycles between the target gene and the housekeeping gene, which was defined as ribosomal protein L13 (RPL13).

Exon–exon spanning primers were designed using the NCBI Primer‐BLAST tool and reference sequences as described in Table 1. For primer validation, single peak melt curves were confirmed by qPCR using either cDNA derived from BHK1 cells or primary hamster cells. The product base pair size was analyzed using a 3% agarose gel. After confirming a single product of the correct size, the product was isolated from the gel band using Monarch DNA gel extraction kit (New England Biolabs, T1020S) following the manufacturer's instructions, sequenced, and aligned with the target sequence using *Geneious Prime*. Primer efficiency was tested by qPCR with serial dilutions of cDNA. Only primers with an efficiency between 90% and 110% were considered efficient.

### 4.8 Serology

#### 4.8.1 RBD‐Specific Enzyme‐Linked Adsorption Assay (RBD‐ELISA)

The ELISA was performed similarly to the method described in [27], demonstrating high sensitivity and specificity. Binding plates (Greiner, Kremsmünster, Austria) were coated with 100 ng/well of SARS‐CoV‐2 RBD antigen overnight at 4°C in 0.1 M carbonate buffer (1.59 g Na_2_CO_3_ and 2.93 g NaHCO_3_, in 1 L distilled water, pH 9.6). The plates were then blocked for 1 h at 37°C using 5% skim milk in phosphate‐buffered saline (PBS). Serum samples were diluted 1:100 in Tris‐buffered saline with Tween 20 (TBST) and incubated on the coated wells for 1 h at 37°C. A multispecies conjugate (SBVMILK; obtained from ID Screen Schmallenberg virus Milk Indirect ELISA; IDvet) diluted 1/80 was applied. Following the addition of tetramethylbenzidine (TMB) substrate (IDEXX), ELISA readings were taken at a wavelength of 450 nm using a Tecan instrument (Tecan Group Ltd., Männedorf, Switzerland), resulting in optical density (OD450 nm) values. Between each step, the plates were washed three times with TBST. For each run, the same defined two replicates of negative (NC) and positive (PC) control sera were included.

### 4.9 Surrogate ELISA

SARS‐CoV‐2 neutralizing antibodies were detected in tissue homogenates and serum using the cPass SARS‐CoV‐2 Neutralization Antibody Detection kit (GeneScript, REFL00847) according to the manufacturer's instructions. Serum samples and controls were diluted 1:10; nasal tissue homogenates were diluted 1:1 in sample buffer. Samples were mixed with HRP‐conjugated SARS‐CoV‐2 RBD and incubated for 30 min at 37°C. The mixture was added to a 96‐well plate coated with hACE2 proteins and incubated for 15 min at 37°C. After washing, the TMB substrate was added, and the reaction was incubated for 15 min in the dark. The reaction was stopped with the stop solution, and optical density (OD) was measured at 450 nm. Signal inhibition (%) was calculated as 100 × (1 − OD450 sample/OD450 negative control). A cut‐off of 30% inhibition was applied.

#### 4.9.1 Virus Neutralization Test

The in vitro assessment of the live virus neutralizing potential of serum samples was conducted on Vero E6 cells (Vero C1008) using 96‐well plates (Greiner). Sera were diluted twofold in triplicate and mixed with an equal volume of medium containing 100 TCID_50_ SARS‐CoV‐2 XBB.1.5 per well. After 1 h incubation of the sample‐virus mixture at 37°C, 100 µL of trypsin‐treated cells (trypsinized cells from a confluent 75 cm^2^ cell culture flask in 50 mL DMEM with 2% penicillin/streptomycin) were added to each well. After 3 days of incubation at 37°C, the presence of CPE was evaluated using a standard optical transmission microscope. Neutralization was defined as the absence of CPE. Neutralization titers of three replicates were calculated as geometric means (reciprocal value) given as virus neutralization titer 100 (VNT100).

### 4.10 Detection of SARS‐CoV‐2 Spike Specific Total Antibodies and IgA by ELISA

SARS‐CoV‐2 XBB.1.5 Spike protein specific IgA and total nonisotype specific antibodies (total Ig) were detected in serum and supernatants of homogenates from hamster conchae and lung, adapting a protocol described in [10]. ELISA plates (96‐well, flat‐bottom; Nunc MaxiSorp) were coated with 100 µL of 1.5 µg·mL^−1^ SARS‐CoV‐2 XBB.1.5/Omicron Spike Trimer Protein (Acro Biosystems) in PBS overnight at 4°C. The following day, plates were washed three times with PBS supplemented with 0.05% Tween 20 (PBS‐T) and incubated with 5% milk in PBS (blocking buffer) for 1 h at RT to block unspecific binding. Organ homogenates were centrifuged at 4000×*g* for 5 min. For IgA ELISA, the organ supernatants were diluted 1:30, and serum was diluted 1:4000 in blocking buffer, before adding 50 µL of the diluted samples to the plates. For the total Ig ELISA, samples were serially diluted threefold in blocking buffer, starting at 1:10 dilution for supernatants and 1:100 dilution for serum. Samples were incubated in the plates for 2 h at RT, then washed three times before adding 50 µL of 1:50‐diluted biotinylated anti‐hamster IgA detection antibody (Brookwood Biomedical, #sab3002a, LOT 14.004) or 1:200‐diluted biotinylated anti‐hamster IgG/M/A (Brookwood Biomedical, #sab3002, LOT:010.000). Following a 2 h (IgA) or 90 min (total Ig) incubation at RT, plates were washed three times and 50 µL of High‐Sensitivity NeutrAvidin HRP conjugate (Thermo Fisher Scientific) was added for 30 min at RT. The plates were washed three times, and 50 µL of 1‐Step Ultra TMB ELISA substrate solution (Thermo Fisher) was added. After 5 min, the reaction was stopped by adding an equal volume of 2 M sulfuric acid. The plates were read for absorbance at 450 nm and 570 nm on a Tecan Infinite M200 Pro microplate reader. Extinction at 570 nm was subtracted as background. The effective dilution to reach 50% of the maximal extinction (ED50) for each sample was determined using a four‐parameter nonlinear regression curve fit in GraphPad Prism v.9.

### 4.11 Testing Isotype Specificity of Anti‐Hamster IgA Antibody

Isotype specificity of two batches of anti‐Syrian hamster IgA antibody was tested by ELISA (Figure S7): ELISA plates (96‐well, flat‐bottom; Nunc MaxiSorp) were coated with fourfold serial dilutions of Syrian hamster IgG isotype control (BioLegend, #402002) in PBS starting at 2 mg/mL overnight at 4°C. The following day, the plates were washed three times with PBS‐T and blocked with 5% milk in PBS for 2 h at RT. Afterwards, biotinylated anti‐hamster IgA detection antibody (Brookwood Biomedical, LOT 14.003 and 14.004) was diluted 1:125, and anti‐hamster IgG (BIOZOL Diagnostica; positive control) was diluted 1:500 and added for 1 h at RT. Biotinylated anti‐human CD3 (UCTH1, BioLegend) was diluted 1:125 and used as an unspecific, negative control. Following the three washing steps, High‐Sensitivity NeutrAvidin HRP conjugate (Thermo Fisher Scientific) was added at a 1:10,000 dilution for 30 min. After three additional washing steps, the signal was developed by adding TMB‐Ultra for 5 min. The reaction was stopped by adding equal parts 20% H_2_SO_4_. The plates were read for absorbance at 450 and 570 nm on a Tecan Infinite M200 Pro microplate reader. Extinction at 570 nm was subtracted as background. Based on the test results, only batch 14.004 was used for further analysis of IgA.

### 4.12 Detection of SARS‐CoV‐2 Specific T Cells With Flow Cytometry

Cells were harvested from lungs as described in [48] and stained with Cell Trace Far Red (Thermo Fisher), prediluted 1:1000 in prewarmed PBS, for 20 min at 37°C. Afterwards, cells were washed three times with PBS. For the stimulation, 1,5 × 10^6^ cells per well were transferred into a sterile U‐bottom plate in RPMI complete. Single cell suspensions were either stimulated with 1 µg/mL Concanavalin A (ConA) as a positive control or 1 µg/mL Spike with 0.5 µg/mL PepMix SARS‐CoV‐2 (JPT) to investigate SARS‐CoV‐2 peptide‐specific responses. A negative control was stimulated with DMSO only. Following 3 days of incubation at 37°C, cells were transferred into a V‐bottom plate for cell surface receptor staining. Cells were washed once with PBS before staining with Zombie Violet fixable viability dye (BioLegend) for 20 min at 4°C in the dark. After two washing steps with Cell staining buffer, unspecific antibody binding was blocked with TruStain FcX (anti‐mouse CD16/32) solution (BioLegend) for 5 min at 4°C in the dark. Cell surface receptors were stained using a freshly prepared antibody cocktail for 20 min at 4°C in the dark. Antibodies were previously established for Syrian hamsters [49]. Details of the antibody clones, fluorochromes, and dilutions are provided in Table S2. Pan‐T cell antibody was freshly labeled using Zenon Mouse IgG2a Labeling Kit, Alexa Fluor 488 (Invitrogen), following the manufacturer's instructions. For final fixation, cells were washed twice and resolved in 4% paraformaldehyde (PFA) for 30 min at RT. protected from light. Cells were then reconstituted in PBS and stored at 4°C in the dark until acquisition on a BD SymphonyA3 flow cytometer using DIVA Version 9.0.1 software, and analyzed using FlowJo Version 10.5.3. The percentage of SARS‐CoV‐2 reactive T cells was determined by subtracting the percentage of proliferated cells in the unstimulated control from that in the stimulated sample.

### 4.13 Histopathology

For histopathology, the left lung lobe was processed as described [10]. Briefly, the left lung lobe was carefully removed, immersion‐fixed in 10% neutral buffered formalin, embedded in paraffin, and 2–3 µm sections were stained with hematoxylin and eosin (HE). Consecutive sections were processed for immunohistochemistry (IHC) using the standardized procedures of the avidin–biotin–peroxidase complex (ABC) method. A primary antibody against the SARS‐CoV nucleocapsid protein was applied overnight at 4°C (Rockland, 200‐401‐A50, 1:3000), and the secondary antibody, a biotinylated goat anti‐rabbit antibody, was applied for 30 min at RT (Vector Laboratories, Burlingame, CA, USA, 1:200). As a negative control, consecutive sections were labelled with an irrelevant antibody (Glial Fibrillary Acidic Protein [GFAP], Abcam, ab16997, 1:600). In each run, an archived control slide from a SARS‐CoV‐2‐infected Syrian hamster was included. All slides were scanned with a Hamamatsu S60 scanner and analyzed by a trained pathologist (T.B.) and reviewed by a certified pathologist (A.B.) in a blinded manner using NDPview.2 plus software (Version 2.8.24, Hamamatsu Photonics, K.K., Japan). The lung tissue was evaluated using a 500 × 500 µm grid, and the extent of pneumonia‐associated consolidation was recorded as a percentage of the affected lung fields. Furthermore, the lung was examined for the presence of SARS‐CoV‐2‐characteristic lesions described for hamsters. These included intra‐alveolar, interstitial, peribronchial, and perivascular inflammatory infiltrates, alveolar edema, necrosis of the bronchial epithelium, diffuse alveolar damage, vasculitis, activation of endothelium with immune cell rolling, as well as bronchial epithelial and pneumocyte type 2 hyperplasia. Following IHC, the distribution of virus antigen was graded on an ordinal scale. The scores were as follows: 0 = no antigen, 1 = focal, affected cells/tissue <5% or up to 3 foci per tissue; 2 = multifocal, 6%–40% affected; 3 = coalescing, 41%–80% affected; 4 = diffuse, >80% affected. The target cell was identified based on its morphological characteristics.

### 4.14 Biosafety Statement

All experiments with infectious SARS‐CoV‐2 were performed in enhanced biosafety level 3 (BSL3) containment laboratories at the Friedrich‐Loeffler‐Institut (FLI), Greifswald‐Insel Riems, Germany. The standard operating procedures of BSL3 facilities were approved by the relevant authorities. All personnel received relevant training before commencing work in BSL3 laboratories.

## Author Contributions

J.S, M.B., and V.T. conceptualized the project. J.K, J.S., D.H., V.T., and N.E. developed the methodology. J.K., J.S., and T.B. conducted formal analysis. J.K., J.S., T.B., N.J.H., L.U., A.B., G.T.B., N.E., B.T., and D.H. conducted investigations. J.K., J.S., and T.B. curated data. J.K. and J.S. wrote the original draft of the manuscript. T.B., N.J.H., A.B., L.U., G.T.B., N.E., V.T., A.D., D.H., M.B., B.C, and J.S. reviewed and edited the manuscript. J.K., J.S., and T.B. performed visualization. J.S., M.B., D.H., and B.C. supervised the project. V.T. and M.B. administered the project. M.B. acquired funding. All authors critically reviewed and agreed to publish this version of the manuscript.

## Funding

The work was funded by RocketVax AG.

## Ethics Statement

All hamster experiments were evaluated by the responsible ethics committee of the State Office of Agriculture, Food Safety and Fishery in Mecklenburg–Western Pomerania (LALLF M‐V) and gained governmental approval under registration number LVL MV TSD/7221.3‐1‐040/22.

## Conflicts of Interest

Related to this work, the University of Bern has filed a patent application for the use of OTS‐300 as a vaccine. In this application, J.S., G.T.B., B.S.T., N.J.H., L.U., N.E., D.H., M.B., and V.T. are named as inventors. The University of Bern and the Friedrich‐Loeffler‐Institut are collaborating with RocketVax AG for the development of OTS vaccines and receive funding for research. VT is consulting for RocketVax AG. The remaining authors declare no conflicts of interest.

## Supporting information




**Supporting File**: eji70196‐sup‐0001‐SuppMat.pdf.

## Data Availability

The data supporting the findings of this study are available in the Supporting Information materials of this article. Viral genome sequences are accessible under BioProject ID PRJNA 1002985 or as specified in the Materials and Methods section.

## References

[1] D. Mannar , J. W. Saville , C. Poloni , et al., “Altered Receptor Binding, Antibody Evasion and Retention of T Cell Recognition by the SARS‐CoV‐2 XBB.1.5 Spike Protein,” Nature Communications 15, no. 1 (2024): 1854. 10.1038/s41467-024-46104-2 PMC10904792 38424106

[2] R. Link‐Gelles , A. A. Ciesla , L. E. Roper , et al., “Early Estimates of Bivalent mRNA Booster Dose Vaccine Effectiveness in Preventing Symptomatic SARS‐CoV‐2 Infection Attributable to Omicron BA.5‐ and XBB/XBB.1.5‐Related Sublineages among Immunocompetent Adults—Increasing Community Access to Testing Program, United States, December 2022‐January 2023,” Morbidity and Mortality Weekly Report 72, no. 5 (2023): 119–124. 36730051 10.15585/mmwr.mm7205e1 PMC9927070

[3] C. H. GeurtsvanKessel , D. Geers , K. S. Schmitz , et al., “Divergent SARS‐CoV‐2 Omicron‐Reactive T and B Cell Responses in COVID‐19 Vaccine Recipients,” Science Immunology 7, no. 69 (2022): eabo2202. 35113647 10.1126/sciimmunol.abo2202 PMC8939771

[4] N. Lasrado , et al., “Waning Immunity against XBB.1.5 Following Bivalent mRNA Boosters,” BioRxiv (2023).

[5] P. J. Halfmann , R. Uraki , M. Kuroda , et al., “Transmission and Re‐Infection of Omicron Variant XBB.1.5 in Hamsters,” EBioMedicine 93 (2023): 104677. 37352827 10.1016/j.ebiom.2023.104677 PMC10329098

[6] N. Lasrado , M. Rowe , K. McMahan , et al., “SARS‐CoV‐2 XBB.1.5 mRNA Booster Vaccination Elicits Limited Mucosal Immunity,” Science Translational Medicine 16, no. 770 (2024): eadp8920. 39441905 10.1126/scitranslmed.adp8920 PMC11542980

[7] M. Gagne , B. J. Flynn , S. F. Andrew , et al., “Mucosal Adenovirus Vaccine Boosting Elicits IgA and Durably Prevents XBB.1.16 Infection in Nonhuman Primates,” Nature Immunology 25, no. 10 (2024): 1913–1927. 39227514 10.1038/s41590-024-01951-5 PMC11436372

[8] J. Tang , C. Zeng , T. M. Cox , et al., “Respiratory Mucosal Immunity Against SARS‐CoV‐2 After mRNA Vaccination,” Science Immunology 7, no. 76 (2022): eadd4853. 35857583 10.1126/sciimmunol.add4853 PMC9348751

[9] Y. Tang , B. P. Boribong , Z. N. Swank , et al., “COVID‐19 mRNA Vaccines Induce Robust Levels of IgG but Limited Amounts of IgA within the Oronasopharynx of Young Children,” Journal of Infectious Diseases 230, no. 6 (2024): 1390–1399. 39253950 10.1093/infdis/jiae450 PMC11646609

[10] J. Schön , G. T. Barut , B. S. Trüeb , et al., “A Safe, Effective and Adaptable Live‐Attenuated SARS‐CoV‐2 Vaccine to Reduce Disease and Transmission Using One‐to‐Stop Genome Modifications,” Nature Microbiology 9, no. 8 (2024): 2099–2112. 10.1038/s41564-024-01755-1 PMC11306094 38997518

[11] X. Liu , W. H. Ng , E. Zusinaite , et al., “A Single‐dose Intranasal Live‐Attenuated Codon Deoptimized Vaccine Provides Broad Protection Against SARS‐CoV‐2 and Its Variants,” Nature Communications 15, no. 1 (2024): 7225. 10.1038/s41467-024-51535-y PMC11347628 39187479

[12] A. Adam , B. Kalveram , J. Y. Chen , et al., “A Single‐dose of Intranasal Vaccination With a Live‐Attenuated SARS‐CoV‐2 Vaccine Candidate Promotes Protective Mucosal and Systemic Immunity,” NPJ Vaccines 8, no. 1 (2023): 160. 37863935 10.1038/s41541-023-00753-4 PMC10589337

[13] J. Trimpert , K. Dietert , T. C. Firsching , et al., “Development of Safe and Highly Protective Live‐Attenuated SARS‐CoV‐2 Vaccine Candidates by Genome Recoding,” Cell Reports 36, no. 5 (2021): 109493. 34320400 10.1016/j.celrep.2021.109493 PMC8289629

[14] M. J. Lett , F. Otte , D. Hauser , et al., “High Protection and Transmission‐Blocking Immunity Elicited by Single‐Cycle SARS‐CoV‐2 Vaccine in Hamsters,” NPJ Vaccines 9, no. 1 (2024): 206. 39472701 10.1038/s41541-024-00992-z PMC11522273

[15] T. Britzke , N. J. Halwe , L. Ulrich , et al., “Live Attenuated SARS‐CoV‐2 Vaccine OTS‐228 Demonstrates Efficacy, Safety, and Stability in Preclinical Model,” NPJ Vaccines 10, no. 1 (2025): 104. 40404620 10.1038/s41541-025-01165-2 PMC12098885

[16] G. Moratorio , R. Henningsson , C. Barbezange , et al., “Attenuation of RNA Viruses by Redirecting Their Evolution in Sequence Space,” Nature Microbiology 2, no. 8 (2017): 17088. 10.1038/nmicrobiol.2017.88 PMC7098180 28581455

[17] G. Nouailles , J. M. Adler , P. Pennitz , et al., “Live‐Attenuated Vaccine sCPD9 Elicits Superior Mucosal and Systemic Immunity to SARS‐CoV‐2 Variants in Hamsters,” Nature microbiology 8, no. 5 (2023): 860–874. 10.1038/s41564-023-01352-8 PMC10159847 37012419

[18] G. Nouailles , E. Wyler , P. Pennitz , et al., “Temporal Omics Analysis in Syrian Hamsters Unravel Cellular Effector Responses to Moderate COVID‐19,” Nature Communications 12, no. 1 (2021): 4869. 10.1038/s41467-021-25030-7 PMC8357947 34381043

[19] V. D. Friedrich , P. Pennitz , E. Wyler , et al., “Neural Network‐assisted Humanisation of COVID‐19 Hamster Transcriptomic Data Reveals Matching Severity States in human Disease,” EBioMedicine 108 (2024): 105312. 39317638 10.1016/j.ebiom.2024.105312 PMC11663782

[20] E. Somogyi , M. Kremlitzka , Z. Csiszovszki , et al., “T Cell Immunity Ameliorates COVID‐19 Disease Severity and Provides Post‐Exposure Prophylaxis After Peptide‐vaccination,” Frontiers in Immunology 14 (2023): 1111629. 36761759 10.3389/fimmu.2023.1111629 PMC9902696

[21] S. Horiuchi , K. Oishi , L. Carrau , et al., “Immune Memory From SARS‐CoV‐2 Infection in Hamsters Provides Variant‐Independent Protection but Still Allows Virus Transmission,” Science Immunology 6, no. 66 (2021): eabm3131. 34699266 10.1126/sciimmunol.abm3131

[22] J. M. Adler , R. Martin Vidal , C. Langner , et al., “An Intranasal Live‐Attenuated SARS‐CoV‐2 Vaccine Limits Virus Transmission,” Nature Communications 15, no. 1 (2024): 995. 10.1038/s41467-024-45348-2 PMC10837132 38307868

[23] H. R. Wagstaffe , R. S. Thwaites , A. Reynaldi , et al., “Mucosal and Systemic Immune Correlates of Viral Control After SARS‐CoV‐2 Infection Challenge in Seronegative Adults,” Science Immunology 9, no. 92 (2024): eadj9285. 38335268 10.1126/sciimmunol.adj9285

[24] V. Fumagalli , M. Ravà , D. Marotta , et al., “Antibody‐Independent Protection Against Heterologous SARS‐CoV‐2 Challenge Conferred by Prior Infection or Vaccination,” Nature Immunology 25, no. 4 (2024): 633–643. 38486021 10.1038/s41590-024-01787-z PMC11003867

[25] L. Beeker , T. Obadia , E. Bloch , et al., “Correlates of Protection Against Symptomatic COVID‐19: The CORSER 5 Case‐Control Study,” Open Forum Infectious Diseases 12, no. 1 (2025): ofaf006. 39872812 10.1093/ofid/ofaf006 PMC11770277

[26] K. McMahan , J. Yu , N. B. Mercado , et al., “Correlates of Protection Against SARS‐CoV‐2 in Rhesus Macaques,” Nature 590, no. 7847 (2021): 630–634. 33276369 10.1038/s41586-020-03041-6 PMC7906955

[27] K. Wernike , A. Aebischer , A. Michelitsch , et al., “Multi‐Species ELISA for the Detection of Antibodies Against SARS‐CoV‐2 in Animals,” Transboundary and Emerging Diseases 68, no. 4 (2021): 1779–1785. 33191578 10.1111/tbed.13926 PMC7753575

[28] K. Wernike , C. Mehl , A. Aebischer , et al., “SARS‐CoV‐2 and Other Coronaviruses in Rats, Berlin, Germany, 2023,” Emerging Infectious Diseases 30, no. 10 (2024): 2205–2208. 39320234 10.3201/eid3010.241079 PMC11431905

[29] B. Ying , T. L. Darling , P. Desai , et al., “Mucosal Vaccine‐Induced Cross‐Reactive CD8(+) T Cells Protect Against SARS‐CoV‐2 XBB.1.5 Respiratory Tract Infection,” Nature Immunology 25, no. 3 (2024): 537–551. 38337035 10.1038/s41590-024-01743-x PMC10907304

[30] C. Riou , R. Keeton , T. Moyo‐Gwete , et al., “Escape From Recognition of SARS‐CoV‐2 Variant Spike Epitopes but Overall Preservation of T Cell Immunity,” Science Translational Medicine 14, no. 631 (2022): eabj6824. 34931886 10.1126/scitranslmed.abj6824 PMC9434381

[31] WHO , Interim Recommendations for the Use of mRNA COVID‐19 Vaccines. 2023.

[32] Y. Wu , N. Wu , X. Jia , et al., “Long‐Term Immune Response to Omicron‐Specific mRNA Vaccination in Mice, Hamsters, and Nonhuman Primates,” MedComm (2020) 4, no. 6 (2023): e460. 38107058 10.1002/mco2.460 PMC10724501

[33] R. Uraki , M. Ito , M. Kiso , et al., “An XBB.1.5‐based Inactivated SARS‐CoV‐2 Vaccine Partially Protects Against XBB.1.5 and JN.1 Strains in Hamsters,” NPJ Viruses 3, no. 1 (2025): 7. 40295856 10.1038/s44298-025-00096-y PMC11790961

[34] S. Havervall , U. Marking , J. Svensson , et al., “Anti‐Spike Mucosal IgA Protection Against SARS‐CoV‐2 Omicron Infection,” New England Journal of Medicine 387, no. 14 (2022): 1333–1336. 36103621 10.1056/NEJMc2209651 PMC9511632

[35] J. E. Oh , E. Song , M. Moriyama , et al., “Intranasal Priming Induces Local Lung‐Resident B Cell Populations That Secrete Protective Mucosal Antiviral IgA,” Science Immunology 6, no. 66 (2021): eabj5129. 34890255 10.1126/sciimmunol.abj5129 PMC8762609

[36] F. Zuo , H. Marcotte , L. Hammarström , and Q. Pan‐Hammarström , “Mucosal IgA Against SARS‐CoV‐2 Omicron Infection,” New England Journal of Medicine 387, no. 21 (2022): e55. 10.1056/NEJMc2213153 36416778

[37] S. Winklmeier , H. Rübsamen , C. Özdemir , et al., “Intramuscular Vaccination Against SARS‐CoV‐2 Transiently Induces Neutralizing IgG Rather Than IgA in the Saliva,” Frontiers in Immunology 15 (2024): 1330864. 38375482 10.3389/fimmu.2024.1330864 PMC10875124

[38] S. Sheikh‐Mohamed , B. Isho , G. Y. Chao , et al., “Systemic and Mucosal IgA Responses Are Variably Induced in Response to SARS‐CoV‐2 mRNA Vaccination and Are Associated With Protection Against Subsequent Infection,” Mucosal Immunology 15, no. 5 (2022): 799–808. 35468942 10.1038/s41385-022-00511-0 PMC9037584

[39] J. Declercq , S. Gerlo , S. Van Nevel , et al., “Repeated COVID‐19 mRNA‐Based Vaccination Contributes to SARS‐CoV‐2 Neutralizing Antibody Responses in the Mucosa,” Science Translational Medicine 16, no. 770 (2024): eadn2364. 39441904 10.1126/scitranslmed.adn2364

[40] T. Mao , B. Israelow , M. A. Peña‐Hernández , et al., “Unadjuvanted Intranasal Spike Vaccine Elicits Protective Mucosal Immunity Against Sarbecoviruses,” Science 378, no. 6622 (2022): eabo2523. 36302057 10.1126/science.abo2523 PMC9798903

[41] S. A. Wellford , A. P. Moseman , K. Dao , et al., “Mucosal Plasma Cells Are Required to Protect the Upper Airway and Brain From Infection,” Immunity 55, no. 11 (2022): 2118–2134 e6. 36137543 10.1016/j.immuni.2022.08.017 PMC9649878

[42] S. R. Allie , J. E. Bradley , U. Mudunuru , et al., “The Establishment of Resident Memory B Cells in the Lung Requires Local Antigen Encounter,” Nature Immunology 20, no. 1 (2019): 97–108. 30510223 10.1038/s41590-018-0260-6 PMC6392030

[43] S. J. Choi , D. Kim , J. Y. Noh , et al., “T Cell Epitopes in SARS‐CoV‐2 Proteins Are Substantially Conserved in the Omicron Variant,” Cellular and Molecular Immunology 19, no. 3 (2022): 447–448. 35043006 10.1038/s41423-022-00838-5 PMC8764507

[44] A. Tarke , C. H. Coelho , Z. Zhang , et al., “SARS‐CoV‐2 Vaccination Induces Immunological T Cell Memory Able to Cross‐Recognize Variants From Alpha to Omicron,” Cell 185, no. 5 (2022): 847–859 e11. 35139340 10.1016/j.cell.2022.01.015 PMC8784649

[45] S. Shou , M. Liu , Y. Yang , et al., “Animal Models for COVID‐19: Hamsters, Mouse, Ferret, Mink, Tree Shrew, and Non‐Human Primates,” Frontiers in Microbiology 12 (2021): 626553. 34531831 10.3389/fmicb.2021.626553 PMC8438334

[46] World Health Organization (WHO) Molecular Assays to Diagnose COVID‐19: Summary Table of Available Protocols, https://www.who.int/docs/default‐source/coronaviruse/real‐time‐rt‐pcr‐assays‐for‐the‐detection‐of‐sars‐cov‐2‐institut‐pasteur‐paris.pdf, 2020.

[47] K. Wernike , B. Hoffmann , D. Kalthoff , P. König , and M. Beer , “Development and Validation of a Triplex Real‐time PCR Assay for the Rapid Detection and Differentiation of Wild‐type and Glycoprotein E‐deleted Vaccine Strains of Bovine Herpesvirus Type 1,” Journal of Virological Methods 174, no. 1‐2 (2011): 77–84. 21458493 10.1016/j.jviromet.2011.03.028

[48] B. Corleis , D. Hoffmann , S. Rauch , et al., “Efficacy of an Unmodified Bivalent mRNA Vaccine Against SARS‐CoV‐2 Variants in Female Small Animal Models,” Nature Communications 14, no. 1 (2023): 816. 10.1038/s41467-023-36110-1 PMC9924835 36781853

[49] E. Kaufmann , N. Khan , K. A. Tran , et al., “BCG Vaccination Provides Protection Against IAV but Not SARS‐CoV‐2,” Cell Reports 38, no. 10 (2022): 110502. 35235831 10.1016/j.celrep.2022.110502 PMC8858710

