# Supplementary material for: Adapted Live SARS‐CoV‐2 Vaccine Elicits Rapid Mucosal Immunity, Protects From Disease, and Reduces Shedding of XBB.1.5
Source: Eur J Immunol. 2026 May 4;56:e70196. doi: 10.1002/eji.70196 (PMC13139754; doi:10.1002/eji.70196)
Supplement: Supplementary file 1 — Supporting File: eji70196‐sup‐0001‐SuppMat.pdf. [file EJI-56-e70196-s001.pdf]

Supplementary Figures

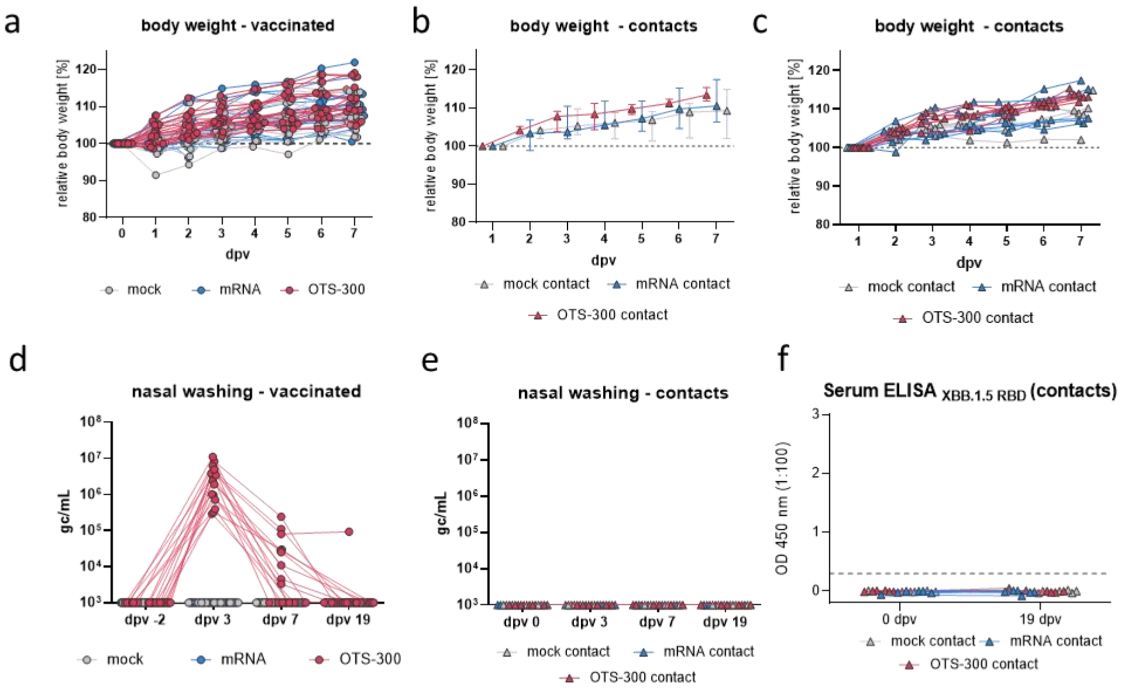

Supplementary Figure 1. Updated OTS-300 is safe and non-transmissible. (a) Individual relative body weight after vaccination. **(b)** Relative body weight (median with interquartile range) of contact animals after vaccination. **(c)** Individual relative body weight of contact animals after vaccination. **(d,e)** SARS-CoV-2 genome copies/mL (gc/mL) in longitudinal nasal wash samples detected by RT-qPCR in **(d)** vaccinated or **(e)** contact animals after vaccination. **(f)** SARS-CoV-2 XBB.1.5 RBD specific serum antibodies in contact animals at d0 and 19 dpv detected with multi species ELISA at 1:100 dilution.

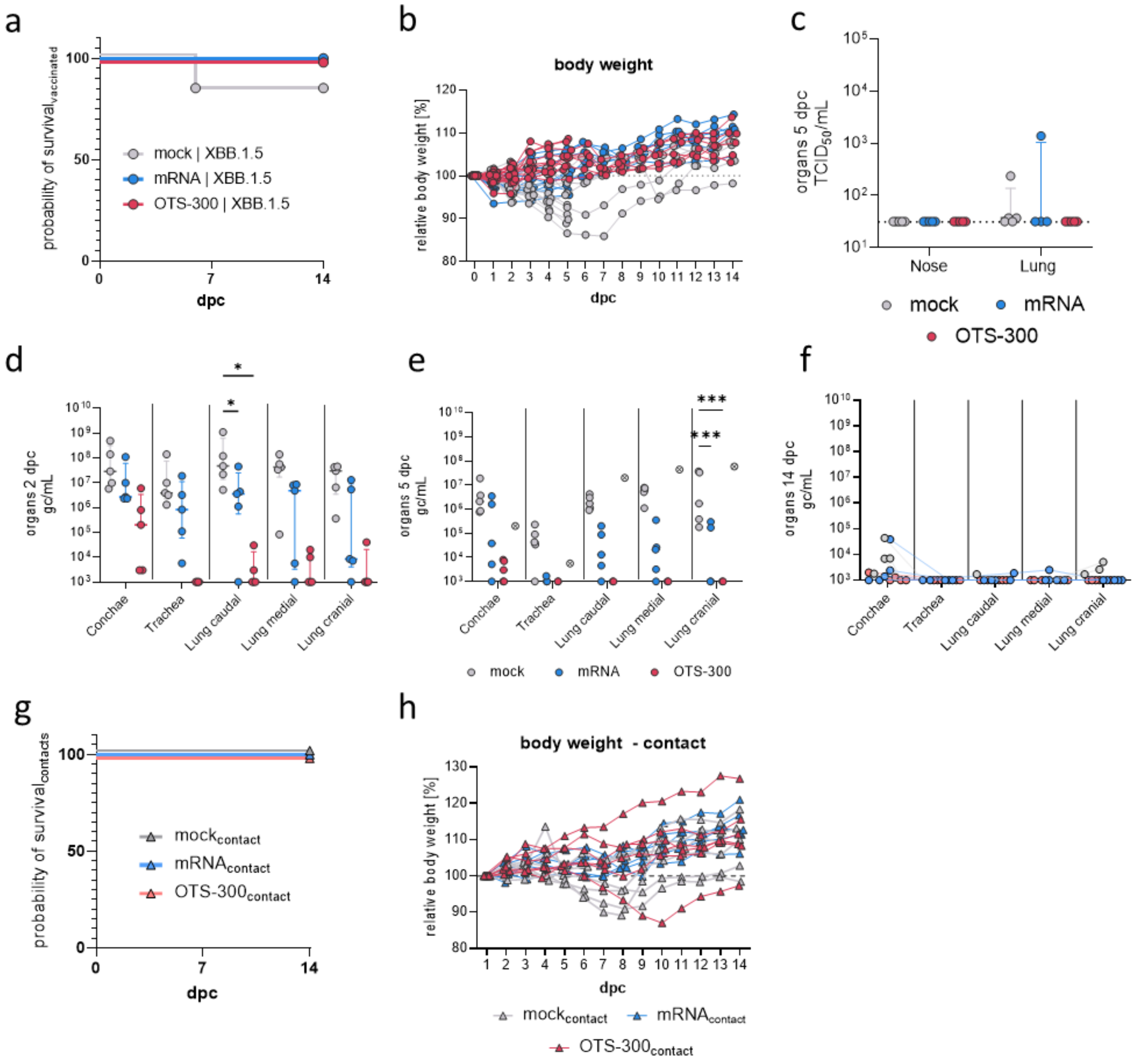

Supplementary Figure 2. OTS-300 efficiently protects against XBB.1.5 challenge infection and reduces transmission. (a) Probability of survival after challenge infection with XBB.1.5 in vaccinated animals. **(b)** Individual body weight after challenge infection in vaccinated animals. **(c)** Individual and median SARS-CoV-2 XBB.1.5 TCID50 per mL with interquartile range in organ samples analyzed tissue culture infectious dose 50% (TCID50) measurement on Vero E6 cells two days post challenge (dpc). (n=5). **(d-f)** SARS-CoV-2 genome copies/mL (gc/mL) as analyzed by RdRp gene-specific RT-qPCR in organs **(d)** 2 dpc, **(e)** 5 dpc, and **(f)** 14 dpc. **(e)** Crossed symbol: Animal died during narcosis 5 dpc. **(g)** Probability of survival in contact animals. **(h)** Individual body weight of contact animals cohoused with vaccinated, infected animals. **(d-f)** $^*P < 0.05$, $^{**}P < 0.01$, $^{***}P < 0.001$, $^{****}P < 0.0001$, 2way ANOVA followed by Tukey's multiple comparisons test.

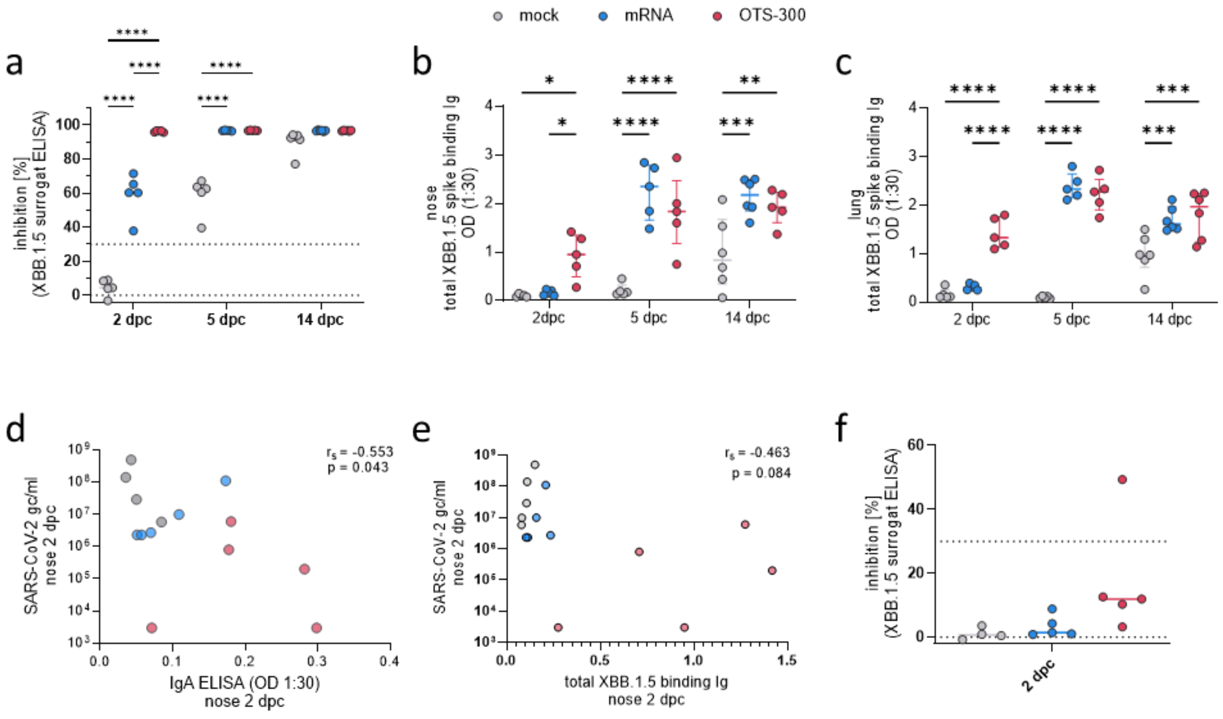

Supplementary Figure 3. OTS-300 induces systemic and mucosal antibody response. (a) Neutralizing capacity of serum samples assessed by XBB.1.5 specific surrogate ELISA. **(b, c)** XBB.1.5 spike specific total Ig measured by ELISA in **(b)** nasal and **(c)** lung samples. **(b-c)***$P < 0.05$, **$P < 0.01$, ***$P < 0.001$, ****$P < 0.0001$, 2way ANOVA followed by Tukey's multiple comparisons test (2 dpc, 5 dpc n= 5; 14 dpc n=6). **(d,e)** Spearman correlation test of viral loads and Spike specific **(d)** IgA or **(e)** total Ig in nasal samples 2 dpc. **(f)** Neutralizing capacity of nasal samples assessed by XBB.1.5 specific surrogate ELISA.

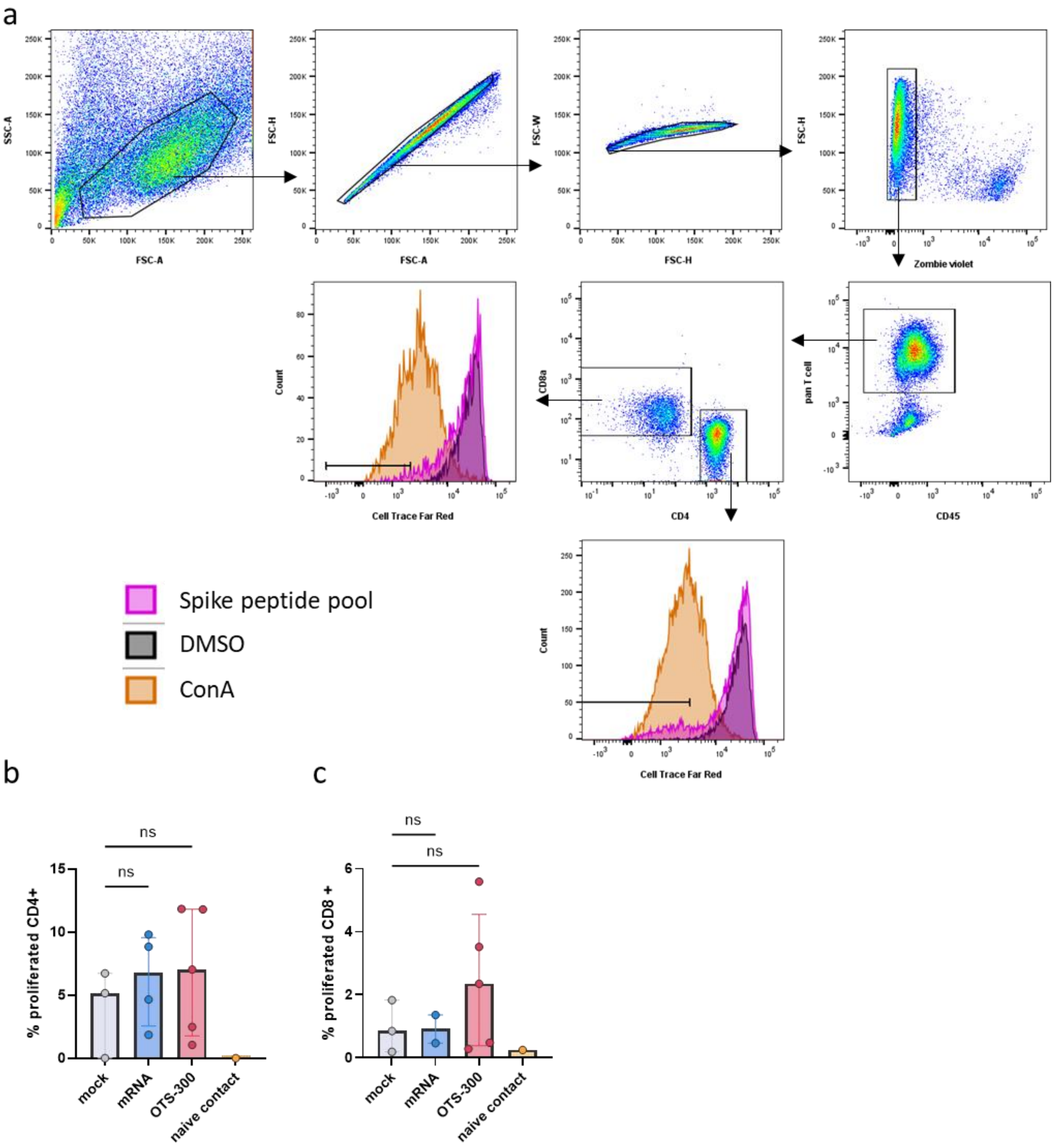

Supplementary Figure 4: Detection of SARS-CoV-2 spike reactive T cells 14 days after challenge infection. (a) Representative image of gating strategy. Lung cells were stained with Cell Trace Far Red and stimulated with SARS-CoV-2 spike peptide pool, Concanavalin A (ConA) or Dimethylsulfoxid (DMSO) for three days prior to staining for flow cytometry. **(b, c)** Frequency of SARS-CoV-2 spike specific, proliferated **(b)** CD4+ and **(c)** CD8+ T cells in lungs 14 dpc with median and interquartile range. Samples with ≤ 900 viable CD4 or CD8 T cells were excluded from analysis. **P* < 0.05 **(b, c)** Kruskal-Wallis test followed by Dunn's multiple comparisons test.

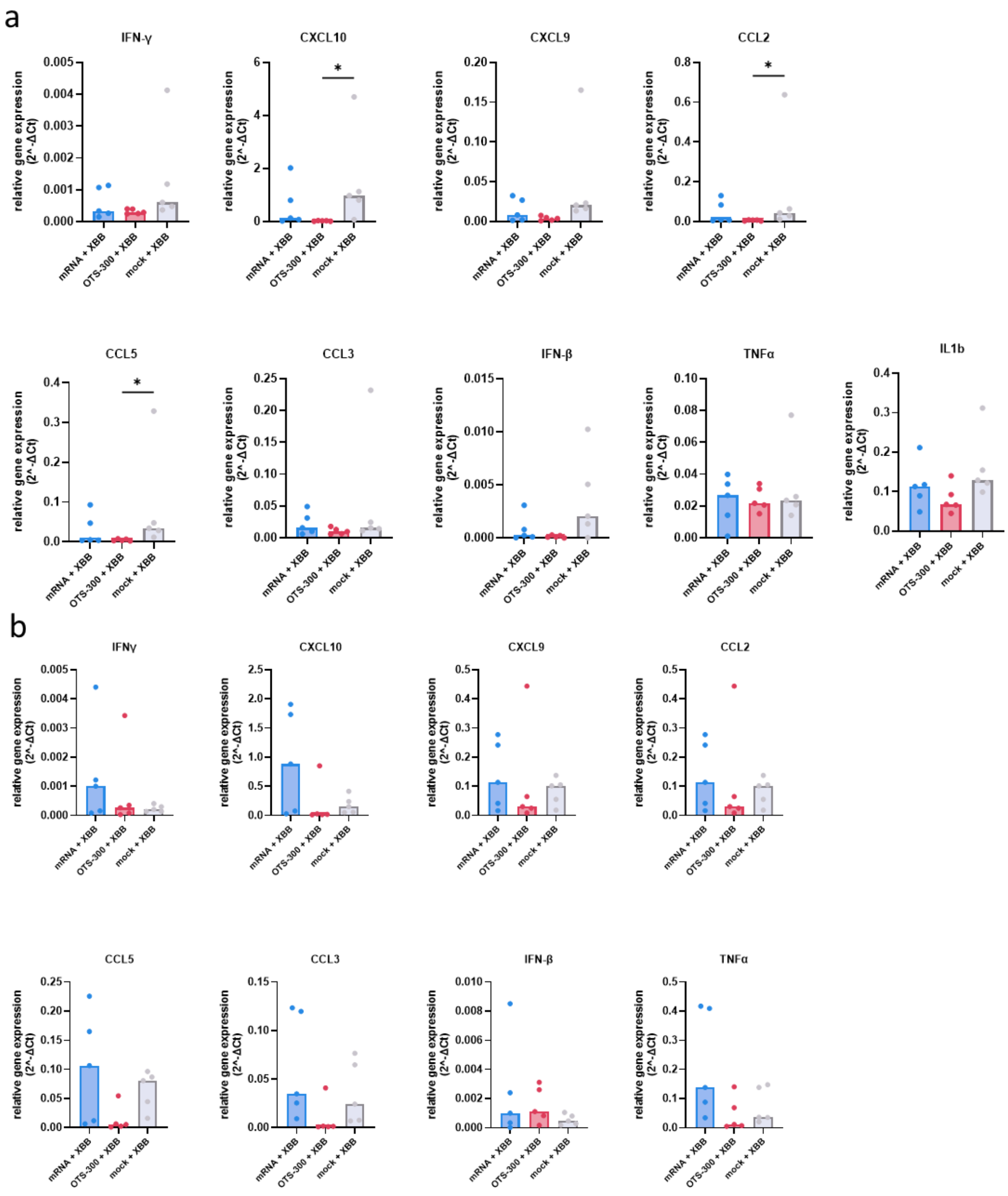

Supplementary Figure 5. OTS-300 reduced inflammatory signalling 2 dpc XBB.1.5 challenge infection. Relative gene expression in **(a)** lung and **(b)** nose 2dpc (n=5). **P* < 0.05, Kruskal-Wallis test followed by Dunn's multiple comparisons test.

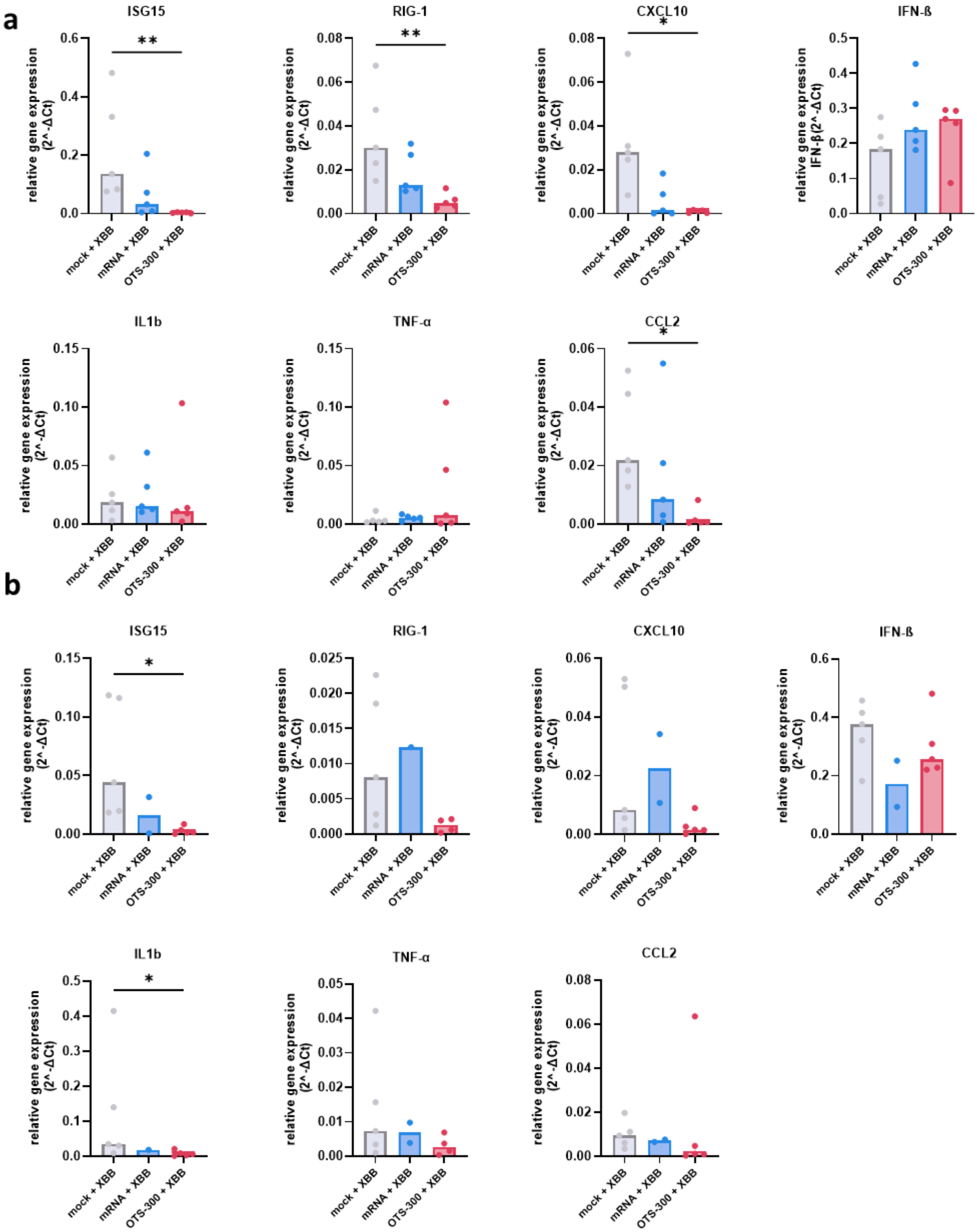

Supplementary Figure 6. OTS-300 reduced inflammatory signalling 5 days post XBB.1.5 challenge infection. Relative gene expression in **(a)** lung and **(b)** nose 5dpc (n=1-5 depending on sample availability). *P < 0.05, Kruskal-Wallis test followed by Dunn's multiple comparisons test.

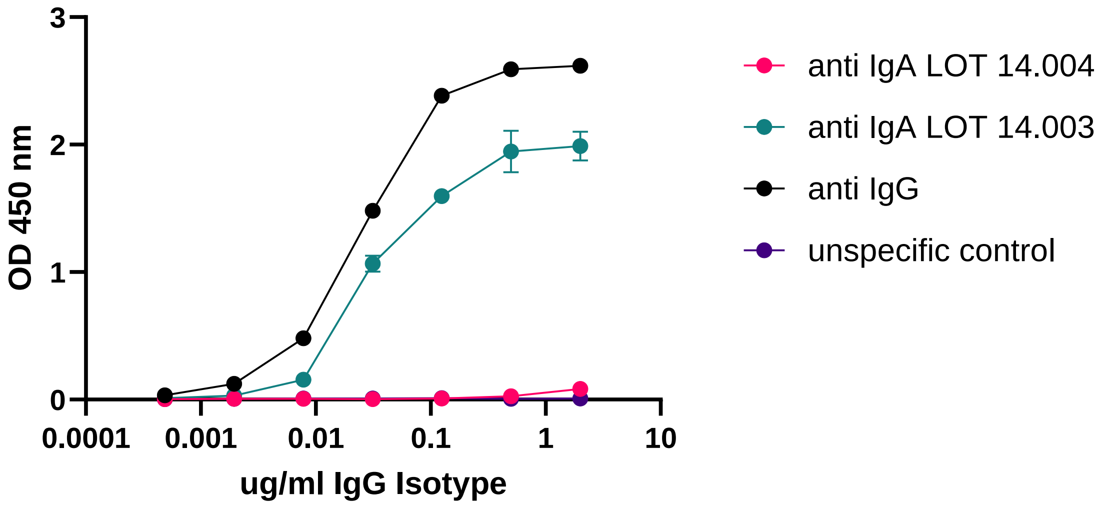

Supplementary Figure 7. Isotype specificity of anti-hamster IgA antibody is batch dependent. Two batches of biotinylated anti- hamster IgA detection antibody (Brookwood Biomedical, LOT 14.003 and 14.004) were tested for cross binding of Syrian hamster IgG Isotype control (Biolegend). Positive control: anti-hamster IgG (BIOZOL Diagnostica); unspecific control: anti anti-human CD3 (UCTH1, Biolegend).

Supplementary Tables

Supplementary Table 1. Primer

**target**	**Forward primer**	**Reverse primer**	**NCBI reference sequence accession number**
RIG-1	AGACTCTGGACCCCACCTAC	AGTCCACAGTAACCTGCGTG	NM_001310553.1
ISG-15	GCCTACAGCCATGACCTGGAA	GACCCTGGCTGATGAGGGTA	XM_013119951.3
IFN-ß1	TCTCCCCATATCCCTGTCCA	TCAGGAGGTCCTCACACTCT	XM_040757177
IFN-γ	AGCCTTGAAGGACAACCAGG	ACCTGAAGGTCATTTACCGGAAT	NM_001281631.1
CXCL-10	TCTGAGTGGGACTCAAGGAATC	ATGATCTCAACACGTGGGCA	NM_001281344.1
CXCL-9	GCACCATCCACTACAGGTCC	CCACTCTTCAGTGTAGCGATGA	XM_040747409.1
TNF	CCCACGTTGTAGCAAACCAC	GTAAACCAGGTACAGCCCGT	XM_005086799
IL-1b	TAGCCAGACTTCCTGTGCAA	AACAGGTCATTCTCATCGCTGT	XM_005068610

CCL-2	GACTCAGGCCAACCCAGAAC	GGAGTTAACGGAGTCTGGCTG	XM_005076967.4
CCL-3	CTGAGCCAGGTGTCATTTTCCTAA	GGTGAAGGCTTCTGGGTTCC	NM_001281338.1
CCL-5	CTCCTTTACACTGCCTCGTGT	TTCCTTCGGGTGACAAAAACGAC	XM_005076936
RPL13	GACTCCCTCCCAAATAGGTGTGAT	ATCCTCAGGGAGATCAGGGGC	XM_005075631

Supplementary Table 2. Antibodies for Flow Cytometry

**molecule**	**species reactivity**	**fluorochrome**	**isotype**	**clone**	**company**	**Cat #**	**dilution**
CD45	mouse	AlexaFluor594	hamster IgG1	HASA25A	Bio-Techne GmbH	NBP2-60909	1:100
CD4	mouse	BB700	Rat LEW/ Lewis IgG2b, κ	GK1.5	BD	745922	1:100
CD8b	rat	PE	Mouse / IgG1, kappa	eBio341 (341)	Invitrogen	12-0080-82	1:100
T Cell	hamster	AlexaFluor 488	mouse	HAT19A	Kingfisher Biotech	WS0766J-100	1:50 *

*after conjugation following manufacturers manual at a 3:1 molar ratio of Fab to antibody target.

Kits

name	manufacturer	Reference number
Applied Biosystems™ High-Capacity cDNA Reverse Transcription Kit	Thermo Fisher SCIENTIFIC<br>Applied Biosystems™	4368814

Zenon™ Mouse IgG2a Labeling Kits , Alexa Fluor 488	Thermo Fisher SCIENTIFIC<br>Invitrogen	Z25102
